# Epigenetic Mechanism Underlying the Development of Polycystic Ovary Syndrome (PCOS)-Like Phenotypes in Prenatally Androgenized Rhesus Monkeys

**DOI:** 10.1371/journal.pone.0027286

**Published:** 2011-11-04

**Authors:** Ning Xu, Soonil Kwon, David H. Abbott, David H. Geller, Daniel A. Dumesic, Ricardo Azziz, Xiuqing Guo, Mark O. Goodarzi

**Affiliations:** 1 Division of Endocrinology, Diabetes and Metabolism, Department of Medicine, Cedars-Sinai Medical Center, Los Angeles, California, United States of America; 2 Medical Genetics Institute, Cedars-Sinai Medical Center, Los Angeles, California, United States of America; 3 Department of Pediatrics, Cedars-Sinai Medical Center, Los Angeles, California, United States of America; 4 Department of Obstetrics and Gynecology, Cedars-Sinai Medical Center, Los Angeles, California, United States of America; 5 Wisconsin National Primate Research Center, University of Wisconsin, Madison, Wisconsin, United States of America; 6 Department of Medicine, David Geffen School of Medicine, University of California Los Angeles, Los Angeles, California, United States of America; 7 Division of Reproductive Endocrinology and Infertility, Department of Obstetrics and Gynecology, David Geffen School of Medicine, University of California Los Angeles, Los Angeles, California, United States of America; University of Muenster, Germany

## Abstract

The pathogenesis of polycystic ovary syndrome (PCOS) is poorly understood. PCOS-like phenotypes are produced by prenatal androgenization (PA) of female rhesus monkeys. We hypothesize that perturbation of the epigenome, through altered DNA methylation, is one of the mechanisms whereby PA reprograms monkeys to develop PCOS. Infant and adult visceral adipose tissues (VAT) harvested from 15 PA and 10 control monkeys were studied. Bisulfite treated samples were subjected to genome-wide CpG methylation analysis, designed to simultaneously measure methylation levels at 27,578 CpG sites. Analysis was carried out using Bayesian Classification with Singular Value Decomposition (BCSVD), testing all probes simultaneously in a single test. Stringent criteria were then applied to filter out invalid probes due to sequence dissimilarities between human probes and monkey DNA, and then mapped to the rhesus genome. This yielded differentially methylated loci between PA and control monkeys, 163 in infant VAT, and 325 in adult VAT (BCSVD P<0.05). Among these two sets of genes, we identified several significant pathways, including the antiproliferative role of TOB in T cell signaling and transforming growth factor-β (TGF-β) signaling. Our results suggest PA may modify DNA methylation patterns in both infant and adult VAT. This pilot study suggests that excess fetal androgen exposure in female nonhuman primates may predispose to PCOS via alteration of the epigenome, providing a novel avenue to understand PCOS in humans.

## Introduction

Polycystic ovary syndrome (PCOS) is a common endocrine disorder, occurring in 7-10% of reproductive aged women [1]. Its heritability has been estimated as high as 0.79 [2]; however, few susceptibility genes have been identified [3]. The pathogenesis of PCOS may be explained by an integrated genetic and epigenetic model [4], with environmental factors contributing to the development of PCOS by modifying the effects of susceptibility genes [5].

PCOS-like phenotypes are produced by prenatal androgenization (PA) of female mammals including rhesus monkeys, sheep, rats and mice, suggesting that the intrauterine environment may play a role in the etiology of PCOS [6]. Fetuses exposed to elevated androgens *in utero* later develop PCOS-like phenotypes as adults, including hyperandrogenism, oligomenorrhea, polyfollicular ovaries, increased adiposity, insulin resistance and impaired insulin secretion [6], [7]. This phenomenon has been formulated as the fetal origins of PCOS hypothesis [7].

The underlying mechanism of fetal origins of PCOS, however, has not been elucidated. Environmental insults during gestation, often related to sex steroids or endocrine disruptors, may predispose to adult disease via epigenetic alterations [8], [9]. Estrogenic exposure, for example, has been shown to reprogram differentiation of target cells via DNA methylation changes during development [10]. Given the crucial effects of epigenetic mechanisms in fetal origins of other metabolic diseases [11], we and others [12] hypothesize that perturbation of the epigenome, through altered DNA methylation during gestation, is a mechanism whereby PA reprograms monkeys to develop PCOS, with transient gestational hyperglycemia accompanying fetal androgen excess in PA monkeys [5] possibly contributing to this process.

The field of epigenetics in PCOS is only now emerging. Epigenetic differences have been demonstrated in PCOS initially in the form of X-chromosome inactivation [13], [14], [15] and genomic instability [16], [17]. Demethylation of the luteinizing hormone receptor (*LHR*) gene also has been detected in a dehydroepiandrosterone (DHEA)-induced mouse model of PCOS [18]. Importantly, our previous finding that total methylation of peripheral blood leukocyte DNA is unaltered in PCOS patients versus matched controls [19] raises the need for site-specific epigenetic studies in physiologically-relevant target tissues. In the current pilot study, we performed a genome-wide site-specific methylation array of visceral adipose tissue (VAT) from PA female rhesus monkeys to test whether the epigenome of this metabolically important fat depot was altered by intrauterine androgen exposure. VAT was first studied due to its close association with metabolic disease [20] and the observation that PA, but not control, female monkeys develop increased VAT that is positively correlated with basal serum insulin levels [21], [22].

## Results

**Figure 1:**
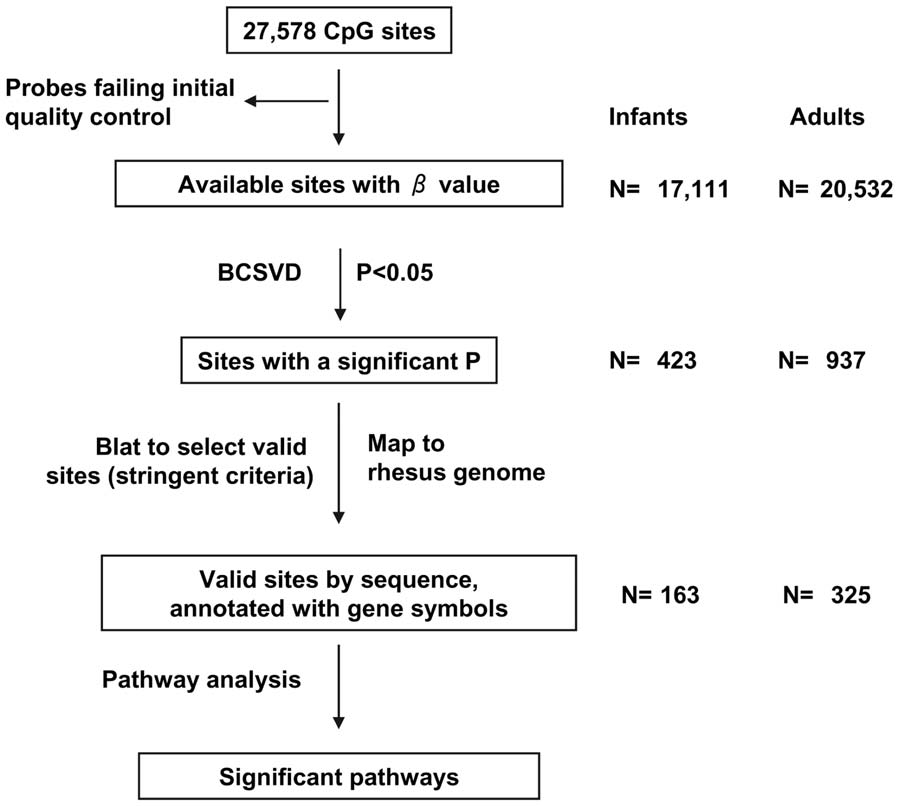
Flow chart of the experimental design and overview of the results.

The flowchart of the epigenetic experimental design is displayed in **Figure 1**. Relevant phenotypic characteristics of infant and adult female monkeys whose dams were injected with testosterone propionate are presented in **Tables 1** and **2**.

**Table 1:** Serum androgen levels of PA infant and control female monkeys.

Monkeys	Testosterone (ng/ml)	Androstenedione (ng/ml)
**Control infants**		
#1	0.07	0.81
#2	0.08	0.92
#3	0.07	1.07
#4	0.05	0.68
#5	NA	NA
**PA infants**		
#1	0.17	2.15
#2	0.10	1.06
#3	0.08	0.69
#4	0.08	2.40
#5	1.33	4.26
#6	0.09	0.54
#7	NA	NA

NA  =  sample not available.

**Table 2:** PCOS phenotype for PA adult monkeys.

Monkeys	Menstrual cycles (mean duration in days)	Ovarian morphology	Testosterone (ng/ml)
**Adult controls**			
#1	Normal (26)	NA	0.12
#2	Normal (28)	NA	0.20
#3	Normal (NA)	NA	0.21
#4	Normal (27)	NA	0.27
#5	Normal (26)	NA	NA
**PA adults**			
#1	Normal (31)	PCO	NA
#2	Intermittent/anovulatory (43)	PCO	0.20
#3	Intermittent/anovulatory (93)	Normal	0.32
#4	Intermittent/anovulatory (70)	PCO	0.48
#5	Intermittent/anovulatory (48)	Normal	0.40
#6	Intermittent/anovulatory (52)	PCO	0.51
#7	Intermittent/anovulatory (120)	PCO	0.29
#8	Intermittent/anovulatory (35)	PCO	0.37

PA female rhesus monkeys exhibit a variety of phenotypes similar to those found in PCOS women. PA monkey phenotypes were refined from those previously described [61], [62]; intermittent/anovulatory cycles were defined as cycle lengths longer than 34 days because 92% of normal female rhesus monkeys have menstrual cycles between 24–34 days [63]; hyperandrogenism was defined as testosterone ≥0.32 ng/ml, which represents the mean plus one standard deviation in circulating basal testosterone levels of control monkeys [62]. PCO: polycystic ovary morphology, determined from photos taken via laparoscope during transabdominal illumination; PCO was defined as the presence of 10 or more ∼1–3 mm follicles per greatest ovarian diameter [64]. NA: not available or not assessed.

### Methylation profiling of infant and adult PA female and control rhesus monkeys

All samples passed the quality check using background-corrected β values (methylation percentages) and built-in controls. Among a total of 27,578 CpG sites, initial quality control generated β values at 17,111 CpG sites for the 12 infant VAT samples, and at 20,532 CpG sites for the 13 adult VAT samples. This quality control pass rate (62–74%) in monkey tissues was predictably slightly lower than that observed in human tissues (∼80%) [23].

No outlier was identified using principal component analysis. The top 5 principal components were selected for further analysis to determine whether potential variance was associated with confounding factors, such as DNA concentration, DNA quality (ODs 260/280), batch/plate effects. No confounding factors were identified (**Table S1**). All 25 samples were therefore retained for further analysis.

Bayesian classification with singular value decomposition (BCSVD) identified 423 sites from infant PA monkeys versus controls and 937 sites from adult PA monkeys versus controls as differentially methylated (P<0.05). After confirming these sites in the rhesus genome, we identified the valid genes (based on sequence comparison of human probes to the monkey genome) with altered methylation levels, resulting in 163 genes from infant data, and 325 from adult data (median methylation levels are given in **Table S2** for infant data and **Table S3** for adult data). The remaining genes were excluded from our analysis due to sequence divergence between species. Our result provides a profile of differential methylation in both infant and adult PA female monkeys.

**Figure 2:**
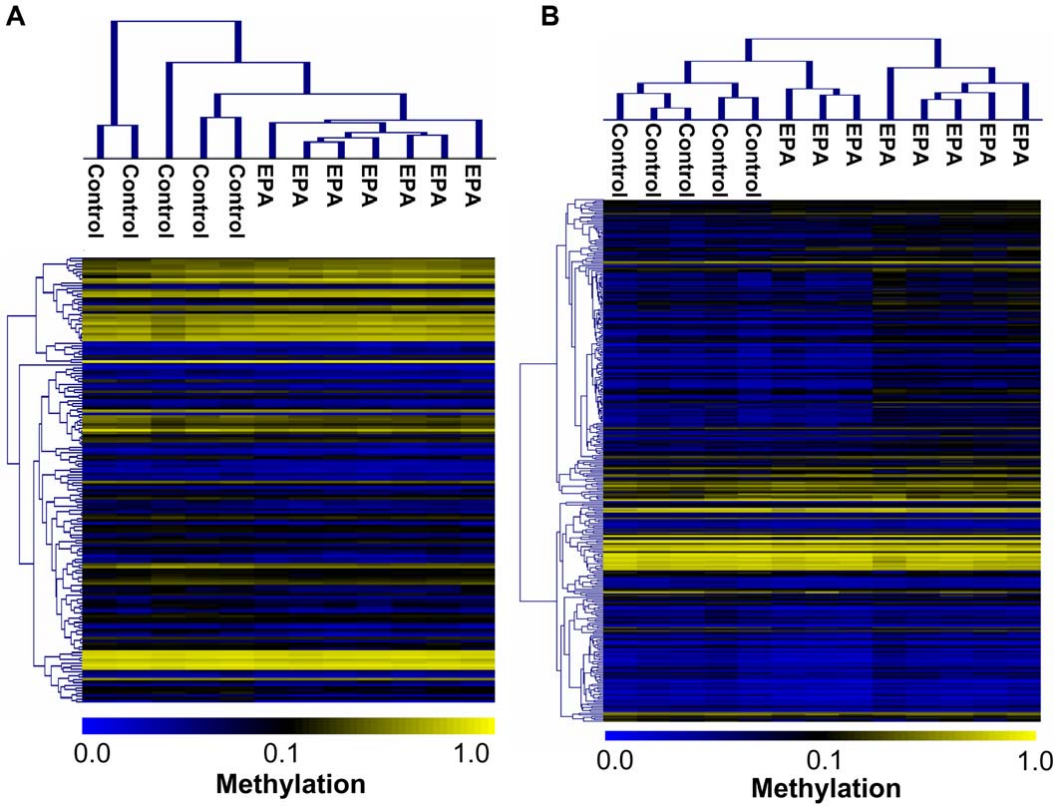
Unsupervised hierarchical clustering. Methylation percentages (β values) at all the significant loci from infant and adult PA monkeys and control monkeys (panel A: 163 loci for infant data; panel B: 325 loci for adult data). Top horizontal bars indicate sample clusters. Scale displays methylation percentage (yellow indicates 1; blue indicates 0).

Hierarchical clustering analyses on the significantly differentially methylated probes are displayed in **Figure 2** for both adult and infant data. Within each age group, the controls and PA samples clustered together. We then compared the two differentially methylated gene sets from infant and adult, and found only two genes with similarly altered methylation in both groups (*FBXO28* and *ZNF512,* see **Table S4** for full names of genes). The same probe of *FBXO28* was hypermethylated in both infant and adult PA female monkeys, and two different probes of *ZNF512* were hypomethylated in both androgenized groups.

### Pathway analysis and potential biological roles

We investigated potential functional pathways and connections between genes with significant differential methylation using the Core analysis algorithm in Ingenuity Pathways Analysis (IPA). The 163 infant genes and 325 adult genes were entered into IPA respectively. The most significant pathways in infant and adult data, respectively, were the anti-proliferative role of TOB (protein encoded by the gene *TOB,* transducer of v-erb-b2 erythroblastic leukemia viral oncogene homolog 2 [*ERBB2*]) in T cell signaling (P = 0.0014), and transforming growth factor-beta (TGF-β) signaling (P = 0.00038) (**Table 3**). The entire pathway diagrams are shown in **Figure S1** (with key to symbols in **Figure S3**), indicating significantly differentially methylated genes in PA monkeys within each pathway. Of note, the anti-proliferative role of TOB pathway contains SMAD4, a participant in TGF-β signaling.

**Table 3:** Significant canonical pathways associated with differentially methylated genes in infants and adults.

Top Canonical Pathways	P-value	Ratio	Genes
**Infant**			
Anti-proliferative Role of TOB in T Cell Signaling	0.0014	3/26 (0.12)	*CDKN1B, PABPC1, SMAD4*
VDR/RXR Activation	0.028	3/78 (0.038)	*CAMP, CYP27B1, CDKN1B*
Methionine Metabolism	0.029	2/31 (0.065)	*CBS, MAT2A*
Complement System	0.034	2/35 (0.057)	*CD55, MASP*
Nucleotide Excision Repair Pathway	0.036	2/35 (0.057)	*ERCC8, POLR2D*
**Adult**			
TGF-β signaling	0.00038	7/83 (0.084)	*BMP2, HOXC9, KRAS, RUNX3, TFE3, TGFB3, TGFBR1*
Axonal Guidance Signaling	0.00072	16/399 (0.04)	*ADAM10, ARPC3, BMP2, EFNB1, EPHA5, ERBB2, ITGA2, KRAS, MYL3, MYL12B, PAK3, PRKAG1, PRKAG2, PRKD1, UNC5B, WNT4*
Tight Junction Signaling	0.0019	9/164 (0.055)	*CPSF3,CSTF3, MAGI2, MYL3, PRKAG1, PRKAG2, SYMPK,TGFB3, TGFBR1*
Polyamine Regulation in Colon Cancer	0.0020	3/17 (0.18)	*KRAS, MXD1, TCF4*
Wnt/B-catenin signaling	0.0024	9/168 (0.054)	*CCND1, RARB, SFRP1, SFRP5, SOX2, TCF4, TGFB3, TGFBR1, WNT4*

The HumanMethylation27 was designed with a focus on cancer, including CpG sites for more than 200 cancer-related and imprinted genes. Thus, cancer-related pathways are expected to be over-represented on the array. In order to determine whether the top pathways identified by IPA were biased by the structure of the array, we conducted pathway analysis by PANTHER, using as the reference gene set the entire list of genes (14,475 genes) on the array, rather than all RefSeq genes. The most significant pathway identified in infant data was “salvage pyrimidine ribonucleotides” (P = 0.014)**,** and the top significant pathway in adult data was again “TGF-β signaling” (P = 0.0061) (**Table S5**). These pathways were not among the most over-represented cancer related pathways on the array (data not shown), suggesting that the pathways over-represented in infant and adult data were not biased by the cancer focus of the array.

We also utilized network analysis in IPA to investigate broader interactions involved in our datasets. The network analysis related all mapped molecules to a higher order interaction over represented by our gene lists. The top network in infant data (**Figure S2A, Table S6**) contained 23 input focus molecules, centered around Akt, ERK1/2, hCG, LH, FSH, Creb and P38MAPK. The top network in adult data (**Figure S2B**, **Table S6**) contained 23 input focus molecules, centered around Akt, ERK1/2, NFêB, TGFBR1, AR, hCG, CCND1, Creb and ERBB2. These networks identified putative central molecules as major functional targets of differential gene methylation in PA female monkeys, from fetal androgen excess and/or gestational hyperglycemia. Many molecules implicated in these networks are involved in regulating adipogenesis and, in agreement with the above pathway analyses, several factors mediating TGF-β signaling were implicated in both infant (ERK1/2, Jnk, SMAD4) and adult (BMP2, ERK1/2, SMAD 2/3, TGFBR1) PA female monkeys.

### Statistical validation and power analysis

To internally validate our findings, we performed leave-one-out cross-validation (LOOCV) analysis based on the model using the 163 differentially methylated genes determined from the infant data. Models based on the 163 genes were able to predict the status of each infant sample with 100% sensitivity and 100% specificity. LOOCV analysis of the 325 differentially methylated genes from the adult data performed similarly in the ability to predict the PA versus control status of each adult sample, consistent with the results of cluster analysis (**Figure 2**). These statistical evaluations affirm the validity of the top differentially methylated genes we have identified.

In addition, we estimated study power of each model (infant and adult) using the generalized likelihood ratio (GLR) method. For the infant model, –lnλ was 8.97, and power  = 1 – Pr(*reject H*_0_)  = 1 – Pr(χ^2^(1)≥ –2lnλ)  = 1 – Pr(χ^2^(1)≥ 17.95) ≈ 1. Similarly for the adult model, –lnλ was 8.25, and thus power  = 1 – Pr(*reject H*_0_)  = 1 – Pr(χ^2^(1)≥ 16.49) ≈ 1. This ensures that analyses within both infants and adults were powered adequately.

### Association between methylation levels and androgen levels in PA female monkeys

We performed regression analyses to determine whether differentially methylated genes identified above in infant and adult samples were associated with their circulating androgen levels (**Table 4**). Methylation levels of *GLO1* and *DDB1* were associated with androgens (testosterone and androstenedione, respectively) in infant PA monkeys. Serum testosterone levels in adult PA monkeys were associated with methylation levels of seven genes: *SUV39H2, OACT2, SEPT9, KCNQ5, RAB6A, PRKD1*, and *OBFC2B*. No associations with androgens were found in controls.

**Table 4:** Significant association between DNA methylation levels and serum androgen levels in PA monkeys.

Probe numbers	Gene Symbol	P value	R value
**Infant**			
26884	*GLO1*	0.041	0.91
3190*	*DDB1*	0.030	−0.97
**Adult**			
11987	*SUV39H2*	0.018	0.97
26822	*OACT2*	0.031	0.88
4479	*SEPT9*	0.032	−0.88
15743	*KCNQ5*	0.041	0.84
10272	*RAB6A*	0.042	0.83
21784	*PRKD1*	0.043	−0.83
2171	*OBFC2B*	0.049	0.81

*All probes were associated with testosterone, except that *DDB1* was associated with androstenedione.

## Discussion

This pilot study suggests that alteration of the epigenome is a mechanism whereby gestational androgen excess, or its consequences, may reprogram fetal female monkeys to develop PCOS-like traits in adulthood. Significant differentially methylated genes identified from infant and adult data are involved in TGF-β signaling, a pathway currently implicated in the pathogenesis of PCOS in women by genetic epidemiologic evidence [24], [25], [26], [27], supporting the potential contributory role of epigenomic perturbation in the etiology of PCOS.

One strength of our study is the selection of VAT, an adipose depot closely linked to metabolic disease [20], which was harvested from PA and control female monkeys to determine whether the epigenome of a specific adipose tissue is modified by *in utero* androgen exposure and its consequences. This is important because DNA methylation likely occurs at tissue-specific differentially methylated regions [28], as evidenced by the tissue-specific differences in methylation levels observed in 12 distinct human tissues [29]. Another strength of this study is the assessment of DNA methylation patterns in both infant and adult VAT. Infant tissues were selected because epigenetic alteration in PA infants would most closely reflect changes from androgen excess *in utero*; adult tissues also were chosen because epigenetic alteration in PA adults would most likely represent postnatal modifications from aging, environmental factors and/or metabolic or reproductive consequences of earlier fetal epigenetic events [30]. In support of this, our data show essentially no overlap between differentially methylated genes in infant and adult PA female monkeys, consistent with monozygotic twins being epigenetically indistinguishable during youth, but showing different patterns of genomic methylated DNA and histone acetylation at older ages [31]. Therefore, an important conclusion of this study is that methylation is plastic; in the adult monkeys, whether epigenetic alteration is a cause or consequence of PCOS-like features cannot be ascertained.

IPA (http://www.geneontology.org) and Panther [32] use different database libraries for pathway enrichment analysis, which may explain the apparently different findings when analyzing infant, but not adult, data. Nevertheless, both programs identified TGF-β signaling as the same top significant pathway in PA adults. In infants, the top pathway identified by IPA was anti-proliferative role of TOB, via *SMAD4*, the co-SMAD that plays a role in transducing the signal of most members of the TGF-β superfamily (**Figure S1A**). The top IPA-generated network for infant PA monkeys also implicated *SMAD4* from altered DNA methylation patterns (**Figure S2A**). Thus, altered methylation in VAT of infants and adults alike appears to disturb signaling by TGF-β superfamily members in PA female monkeys with PCOS-like phenotypes. Given dysfunctional TGF-β signaling as a putative genetically-based mechanism for developing PCOS [24], [25], [26], [27], our findings of altered methylation of *TGFB3, TGFBR1, KRAS, BMP2, TFE3, Runx3* and *Hoxc8*, complement previous studies implicating TGF-β superfamily members or their regulators in the pathophysiology of PCOS, including anti-Müllerian hormone (AMH), inhibin B, growth differentiation factor 9, activin A, follistatin, and fibrillin-3 [24], [25], [26], [27], [33], [34], [35]. Because fibrillins interact with latent TGF-β binding protein, and both fibrillins and follistatin share TGF-β binding domains, both regulate extracellular TGF-β bioactivity [36], [37].

Several of the genes whose differential methylation was associated with androgen levels are involved in cell membrane function and/or structure (*OACT2*, acylation of membrane lysophospholipids; *SEPT9*, cell division/cell membrane cleavage; *RAB6A*, intracellular membrane trafficking [38], [39], [40]). We hypothesize that these membrane-related functions may be important to TGF-β signaling. In support of this, SEPT9 binds to hypoxia-inducible factor-1*α* (HIF-1*α*), which increases transcription of TGF-*β* [41], [42]. Additionally, SUV39H2 binds to and potentiates the effects of SMAD5, which participates in the signaling of the bone morphogenetic proteins, which are members of the TGF-*β* superfamily [43].

In addition, our pilot study provided additional hypothesis-generating results, based on network analysis identifying Akt, ERK1/2, hCG, and Creb (**Figure S2**) as factors central to infants and adults. Akt and ERK1/2 participate in insulin [44] and MAPK signaling pathways (KEGG Pathway Database, http://www.genome.jp/kegg/); genetic variants of *AKT2* have been associated with PCOS in humans [45], while altered expression of MAPK signaling pathway components has been demonstrated in PCOS cumulus cells [46]. It remains to be examined whether epigenetically-mediated altered interactions between these factors and hCG/LH in adipose tissue are the basis by which adiposity in PCOS, as a determinant of abnormal gonadotropin secretion, alters ovarian function [47].

Some, but not all, recent studies argue against prenatal androgenization as a possible developmental programming model for human PCOS. Women from male/female twin pairs were not found to have an increased prevalence of PCOS versus women from same sex twin pairs; however, the overall prevalence of PCOS (3-4%) in that study was lower than reported in most other populations, possibly limiting the power of the study to detect an effect on PCOS [48]. Also, umbilical cord blood androgen levels were not found to be elevated in adolescent girls later diagnosed with PCOS [49], nor in one study were they elevated in the infant offspring of women with PCOS [50]. In contrast, another study noted elevated testosterone levels in umbilical venous blood of females born to PCOS women [51]. Moreover, a study of human fetal blood obtained via cordocentesis found that ∼40% of female fetuses at mid-gestation had testosterone levels in the male range [52]. Further studies are needed to quantify serum androgen levels in the mid-gestational human female fetus, which may itself be the source of androgen excess *in utero* during a critical time of target tissue differentiation [7], [53].

Although experimentally induced fetal androgen excess is an artificial system, the fact remains that PA monkeys (as well as sheep, rats, mice) develop PCOS-like phenotypes, and monkeys can naturally develop PCOS-like traits without apparent intervention [54]. The PA rhesus model enables us to study intrauterine androgen influences on fetus and infant, which is difficult to study in humans. Studying PA rhesus monkeys will be highly informative to understand underlying epigenetic mechanisms of steroid, and possibly hyperglycemic [5], action, including molecular factors and/or pathways altered by prenatal androgen and glucose excess, and to posit pathophysiological processes that may influence the phenotypic expression of PCOS.

Our genome-wide methylation analysis may have missed some key factors or networks since it did not interrogate every gene of the monkey genome (27,578 CpG sites targeted; 14,475 genes studied) and 20–30% of all probes did not pass the initial quality control. Nevertheless, this high throughput methylation assay profiled a large number of genes, most of which would not be considered in a classical candidate approach. Also, not all CpG sites need to be assessed because of correlation of methylation status between sites [55].

In conclusion, this study identified the first specific alterations in the epigenome of PA infant and adult rhesus monkeys, suggesting that fetal androgen excess and its consequences have epigenetic implications for developmental programming of PCOS. While exhibiting virtually no overlap in terms of specific genes differentially methylated, the infant and adult methylation alterations pointed to common pathways of pathogenesis. Epigenetic alterations in PA monkeys may provide a novel avenue to understand the developmental origins and pathophysiology of PCOS in humans.

## Materials and Methods

### Ethics statement

No work with live animals was conducted in this study. Monkey tissues harvested at the Wisconsin National Primate Research Center (WNPRC) were sent to Cedars-Sinai Medical Center for DNA extraction and epigenetic analysis. Some of the adult female rhesus monkey tissues were obtained through the WNPRC's Nonhuman Biological Material Distribution program. The WNPRC principal investigator of the studies that originally obtained the monkey tissues (D.H. Abbott) gave permission for fellow authors to use harvested samples for this study. The care and housing of, and harvesting of tissues from, female rhesus monkeys at both the California National Primate Research Center (CNPRC) and the WNPRC (infants [56] and adults [57], respectively) were fully compliant with the recommendations of the Guide for the Care and Use of Laboratory Animals and the Animal Welfare Act. All protocols were approved before implementation by the Institutional Animal Care and Use Committees at the University of California, Davis and the Graduate School, University of Wisconsin, Madison.

### Samples

The study sample consisted of VAT from 25 female rhesus monkeys at infant or adult stages. The infant (8–9 weeks of age) samples comprised VAT from 5 controls and 7 PA monkeys. The adult (age >17 years) samples consisted of VAT from 5 controls and 8 PA monkeys with phenotypic derangements resembling PCOS. Adult controls were selected to match the age, body weight, and BMI range of the PA females. A detailed description of study design and methodology has been reported previously [6], [56], [57]. Briefly, early gestation-exposed PA female monkeys were produced by daily subcutaneous injections of their dams with 10–15 mg testosterone propionate for 15–40 consecutive days beginning on gestational days 40–44 (total gestation, 165±10 days). VAT was collected after infant and adult monkeys were sacrificed. Monkeys were put through timed injection with ketamine and barbiturate prior to necropsy. During the necropsy protocol, VAT was obtained from omental depots adjacent to the gastrointestional tract. VAT was placed into sterile cryovials, which were immediately placed into liquid nitrogen and transported to a −80°C freezer soon afterwards.

Three of the 15 infant samples previously described [56] were excluded from this study. One control infant was omitted as an outlier due to elevated maternal/fetal basal serum glucose levels in the third trimester, elevated birth weight, and elevated basal serum glucose levels at 2 months postnatal age [5]. Two PA infants were omitted because their mothers had impaired glucose intolerance during pregnancy, which may alter fetal epigenetic patterns [58]. Regarding adult monkeys, of the 34 adults previously described [57] we studied a subset of 13 early-gestation PA monkeys that had undergone necropsy and tissue collection. Some adult PA females died of natural causes and were found dead in their cages. Necrosis was too extensive in those cases for useful organ/tissue harvest. Normal female monkeys were subset driven by need of other investigators or colony management for necropsy at the WNPRC (but without treatment prior to euthanasia).

### Genome-wide DNA methylation analysis

Genomic DNA was extracted from all 25 VAT samples using the ALLPrep kit (Qiagen, Valencia, CA), followed by bisulfite conversion, during which unmethylated cytosine is converted to uracil, and methylated cytosine is not converted. After whole genome amplification, samples were subjected to genome-wide DNA methylation analysis using the Infinium HumanMethylation27 BeadChip (Illumina, San Diego, CA), which employs two different bead types to detect CpG methylation. The U beads bind to unmethylated CpG sites, and the M beads bind to the methylated sites. The match between bead type and the CpG site enables single base extension, incorporating a florescence-labeled ddNTP for detection. Image processing and intensity data extraction were performed according to Illumina's instructions. Methylation status of each interrogated locus is measured as the β value, representing the ratio of fluorescent signals from the methylated probes to total locus intensity (M/(M+U)), ranging from 0 (completely unmethylated) to 100% (completely methylated). This assay simultaneously measures methylation levels at 27,578 CpG sites in or within 1.5 kb of 14,475 genes.

### Quality control

Background-corrected β values and built-in controls were used to evaluate the quality of individual arrays. Sample quality was evaluated via bisulfite conversion efficiency and outliers on histograms of β values. Samples were filtered using the Beadstudio P-values of detection of signal above background.

After the first quality control step, samples were subjected to principal component analysis (PCA) to identify outliers, if any. Infant and adult samples were analyzed separately since they were performed on different dates. After generating eigenvalues, samples were analyzed against the top principal components to determine whether experimental factors such as batch/plate, DNA input (amount and quality) were associated with principal components. Linear regression was performed for continuous variables such as DNA amount and quality (ODs of 260/280), and logistic regression was performed for nominal variables such as batch/plate.

### Unsupervised hierarchical clustering

Cluster analysis was performed to divide samples into groups on the basis of similarities among their DNA methylation profile using TIGR MeV (http://www.tm4.org/mev/) [59]. Infant and adult data sets were clustered separately.

### Bayesian Classification with Singular Value Decomposition (BCSVD)

Two experiments were evaluated: PA infants versus control infants, and PA adults versus control adults. The BCSVD method was utilized to identify significantly differentially methylated probes in the genome-wide methylation data. BCSVD is a practical method to identify genetic determinants in large scale association studies when the sample size is much smaller than the number of markers [60]. In contrast to most current statistical methods, which analyze one genetic variant at a time, this method tests all probes simultaneously in a single test, thus avoiding the multiple testing problem. The BCSVD method achieves dimension reduction by applying singular value decomposition (SVD) to the design matrix in the model. Model fitting was achieved via Markov chain Monte Carlo (MCMC) with Gibbs sampler. Significant probes were selected based on the empirical P values (<0.05) that were calculated using permutation.

### Criteria for experimentally valid probes

Each probe consists of 50 nucleotides matching a CpG site at the end. To handle sequence dissimilarities between human probes and monkey DNA template, we formulated stringent criteria to filter all probes with significant BCSVD P values by performing alignments of the human probes to the monkey genome. These criteria were: 1) the 3′ end of the probe with the CG end had to match between the human probe and monkey DNA, to enable single base extension and thus detection; 2) the number of mismatched nucleotides of alignments was less than or equal to 3 of the 50-nucleotide probe, such that identity was greater than or equal to 94%; and 3) if multiple matches were found among alignments, only the genomic sequence containing the top match with 100% identity was selected to be valid. Those probes that met these criteria were retained for further exploration, and others were removed from the list. Re-analysis revealed that removal of invalid probes did not affect overall results generated with BCSVD.

Next we mapped all statistically significant probes to the rhesus genome using Blat (http://genome.ucsc.edu/cgi-bin/hgBlat), to confirm 1) gene annotations of those probes from the human genome-based array are the same as their gene annotations in the rhesus genome; and 2) probes are located within 1.5 kb upstream of the transcription start site or overlapping the gene body in the rhesus genome, similar to how they were designed for humans in the Infinium arrays. This ensured the human gene annotations for CpG sites were accurate for the monkey genome.

### Statistical validation and power analysis

Within each experiment (infant and adult, respectively), we evaluated the validity of the fitted model with all probes selected as significant (P<0.05, and meeting criteria for experimentally valid) to distinguish PA monkeys from controls, utilizing leave-one-out cross-validation (LOOCV) analysis. LOOCV leaves one sample out at a time, fits the model on the remaining samples, and then uses the model to predict the case/control status of the held out subject. If the fitted model is valid, we can expect a high accuracy rate for its predictions.

The power of each model (infant and adult) was estimated using the generalized likelihood ratio (GLR) under the null hypothesis *H*_0_ : intercept only model versus the alternative *H*_1_ : the model with all selected probes. Power  = 1 – Pr(*reject H*_0_)  = 1 – Pr(χ^2^(1)≥ –2lnλ), where λ is the GLR under *H*_0._ For large sample size, -2lnλ follows a chi-square distribution with one degree of freedom.

### Pathway analysis

The two differentially-methylated gene sets from both infant and adult data were analyzed through the use of Ingenuity Pathways Analysis (IPA) (Ingenuity Systems, Redwood City, CA, www.ingenuity.com). Canonical pathways in the Core analysis identified the pathways from the Ingenuity Knowledge Base library of canonical pathways that were most significant to the data set. The significance of the association between the data set and the canonical pathway was measured in 2 ways: 1) the right tailed Fisher's exact test was used to calculate a P value, and significance was taken as P value <0.05; 2) a ratio of the number of molecules from the data set that map to the pathway divided by the total number of molecules comprising the canonical pathway.

Furthermore, we used the PANTHER (Protein ANalysis THrough Evolutionary Relationships) program. PANTHER can compare input gene lists to any reference gene list, and identify significant pathways that are either over-represented or under-represented by the input list [32]. We used the entire gene list of the HumanMethylation27 chip as a reference set, instead of the human RefSeq. This analysis enabled us to determine whether our results were biased by the array structure.

### Network Analysis

To find networks of highly connected focus genes (input genes), network analysis in the Core analysis was performed in IPA, representing the molecular relationships between gene products. Core networks contained both direct and indirect interactions. Each uploaded gene symbol was mapped to its corresponding gene object (focus gene) in the Ingenuity Pathways Knowledge Base, and focus genes were selected as “seed” elements to generate networks with the highest connectivity to other focus genes. Non-focus genes that were not among our differentially methylated gene lists were also added if they contained links to the network. Networks were scored for the likelihood of finding the focus molecules in that given network. The ranking score for each network was computed based on the right tailed Fisher's Exact Test as the negative log of the probability that the number of focus genes in the network was due to random chance.

### Association analysis between phenotypes and methylation profiles

Association testing was performed using the GoldenHelix (Bozeman, Montana) program. Numeric variables such as androgens were analyzed for correlation with methylation levels using linear regression, corrected for multiple testing including Bonferroni correction and false discovery rate.

## Supporting Information

Figure S1 Diagrams of the significant canonical pathways detected in PA infant (top) and PA adult (bottom) VAT versus age-specific controls. Shaded genes (red) are input molecules with differential methylation. Panel A displays the most significant pathway from infant data:, the antiproliferative role of TOB in T-cell signaling, which includes *PABP1*, *SMAD4*, and *P27KIP1* that are differentially methylated in infants. Panel B displays the most significant pathway from adult data, TGF-β signaling, which includes *TGFB3*, *TGFBR1*, *KRAS*, *BMP2*, *TFE3*, *RUNX3* and *HOXC8* from the list of genes differentially methylated in adults. Arrow-up (red) indicates hypermethylation; arrow-down (blue) indicates hypomethylation relative to controls (also see **Figure S3** for detailed explanation of shapes and relationships). Note: two arrows of *BMP2* indicate that the two probes of this gene were significantly differentially methylated in opposite directions.
(TIF)

Figure S2 Significant networks connecting genes that were significantly differentially methylated in PA VAT samples compared to controls. Panel A displays the most significant network from infant data, centered around Akt, hCG, ERK1/2, LH, FSH, Creb and P38MAPK. Panel B displays the most significant network from adult data, centered around Akt, ERK1/2, NFκB, TGFBR1, ERBB2, hCG, CCND1, Creb and AR. Molecules are represented as nodes, and the biological relationship between two nodes is represented as an edge (line). Shaded factors (red) are input molecules whose genes generated a significant BSCVD P value. Darker shading of filled molecules represents a lower and thus more significant BSCVD P value. Factors in unfilled nodes were not among our differentially methylated list but contained links to the network. Arrow-up (red) indicates hypermethylation; arrow-down (blue) indicates hypomethylation relative to controls (also see **Figure S3** for detailed explanation of shapes and relationships).
(TIF)

Figure S3 Keys to symbols in networks and pathways.
(TIF)

Table S1 Summary of regression analysis to determine whether experimental factors were associated with any principal components in infant data (top table) and adult data (bottom table). P values are displayed in the table.
(DOC)

Table S2 163 significantly differentially methylated genes deemed valid when comparing infant PA and control monkeys. Median and interquartile range (IQR) are presented for control and PA monkeys at each probe. Genes were sorted by BSCVD P values.
(DOC)

Table S3 325 significantly differentially methylated genes deemed valid when comparing adult PA and control monkeys. Median and interquartile range (IQR) are presented for control and PA monkeys at each probe. Genes were sorted by BSCVD P values.
(DOC)

Table S4 Full names of genes in text.
(DOC)

Table S5 PANTHER pathway analysis generated with the Human Methylation27 genes as the reference list.
(DOC)

Table S6 The highest scoring molecule networks in PA infant and adult female monkeys. *  =  involved in TGF-β signaling; **  =  involved in reproduction; ***  =  involved in adipogenesis.
(DOC)
