# Supplementary figures and images for: Epigenetic Mechanism Underlying the Development of Polycystic Ovary Syndrome (PCOS)-Like Phenotypes in Prenatally Androgenized Rhesus Monkeys
Source: PLoS One. 2011 Nov 4;6(11):e27286. doi: 10.1371/journal.pone.0027286 (PMC3208630; doi:10.1371/journal.pone.0027286)

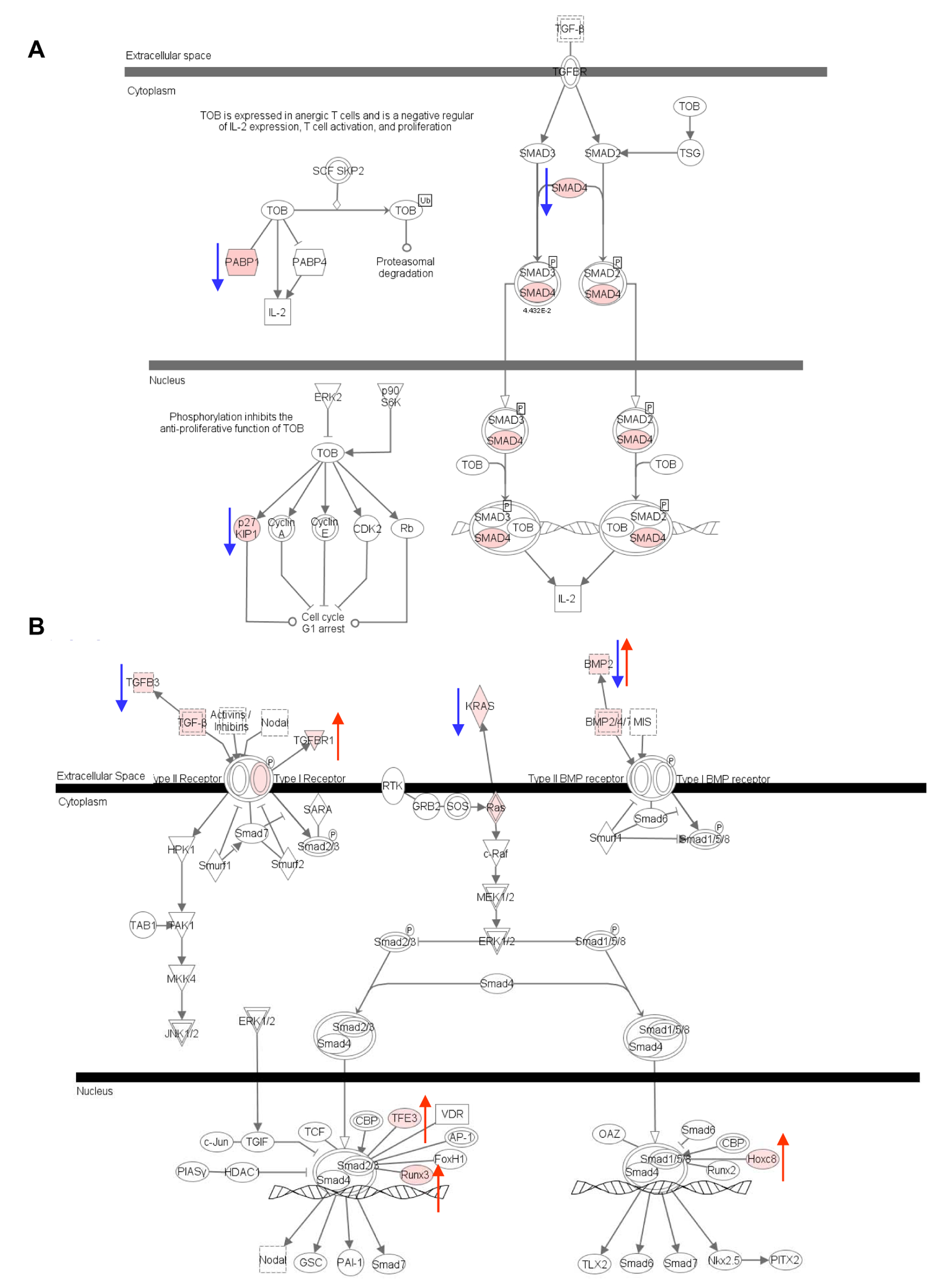

Supplement: Figure S1 — Diagrams of the significant canonical pathways detected in PA infant (top) and PA adult (bottom) VAT versus age-specific controls. Shaded genes (red) are input molecules with differential methylation. Panel A displays the most significant pathway from infant data:, the antiproliferative role of TOB in T-cell signaling, which includes PABP1, SMAD4, and P27KIP1 that are differentially methylated in infants. Panel B displays the most significant pathway from adult data, TGF-β signaling, which includes TGFB3, TGFBR1, KRAS, BMP2, TFE3, RUNX3 and HOXC8 from the list of genes differentially methylated in adults. Arrow-up (red) indicates hypermethylation; arrow-down (blue) indicates hypomethylation relative to controls (also see Figure S3 for detailed explanation of shapes and relationships). Note: two arrows of BMP2 indicate that the two probes of this gene were significantly differentially methylated in opposite directions. (TIF) [file pone.0027286.s001.tif]

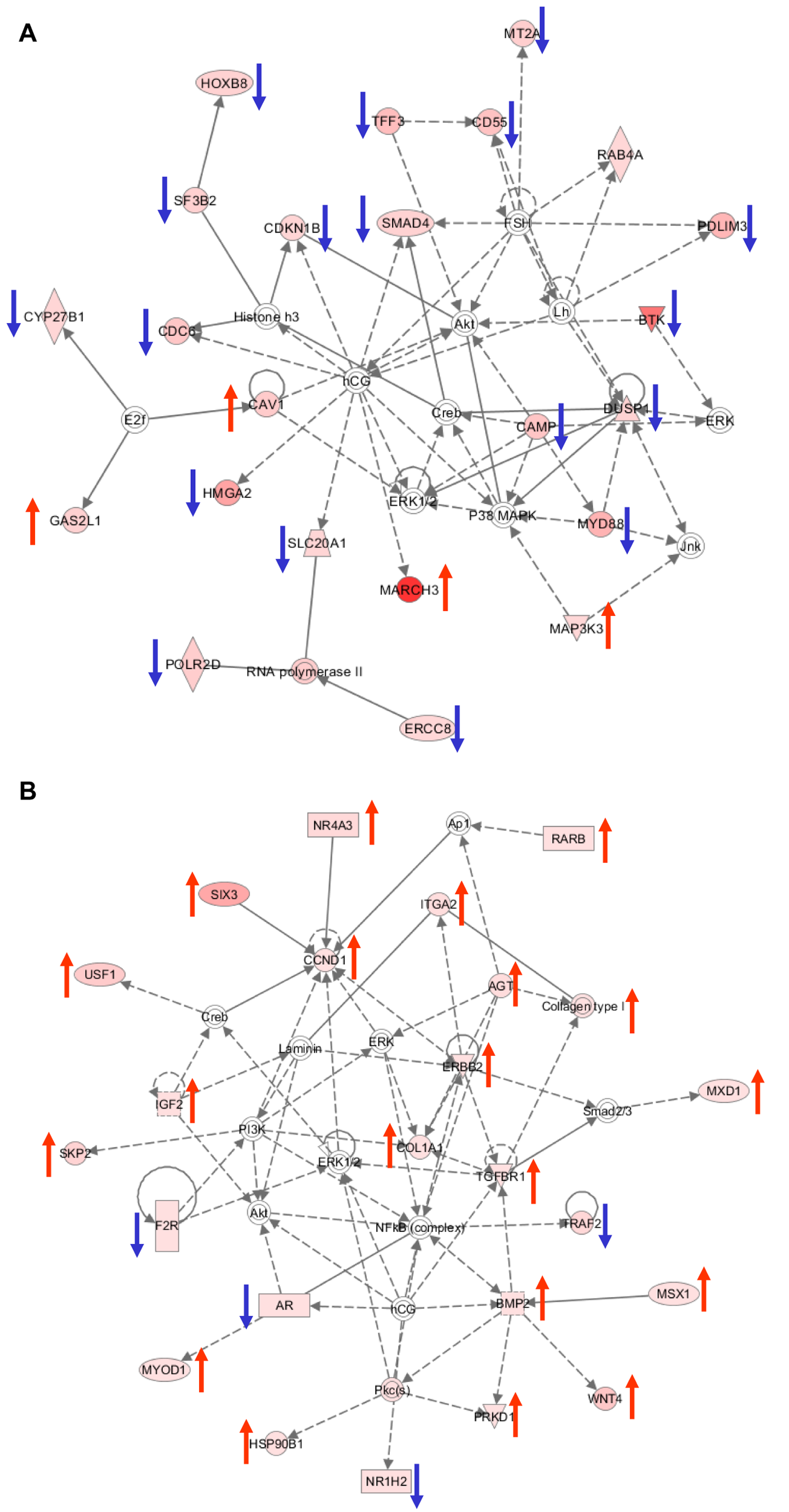

Supplement: Figure S2 — Significant networks connecting genes that were significantly differentially methylated in PA VAT samples compared to controls. Panel A displays the most significant network from infant data, centered around Akt, hCG, ERK1/2, LH, FSH, Creb and P38MAPK. Panel B displays the most significant network from adult data, centered around Akt, ERK1/2, NFκB, TGFBR1, ERBB2, hCG, CCND1, Creb and AR. Molecules are represented as nodes, and the biological relationship between two nodes is represented as an edge (line). Shaded factors (red) are input molecules whose genes generated a significant BSCVD P value. Darker shading of filled molecules represents a lower and thus more significant BSCVD P value. Factors in unfilled nodes were not among our differentially methylated list but contained links to the network. Arrow-up (red) indicates hypermethylation; arrow-down (blue) indicates hypomethylation relative to controls (also see Figure S3 for detailed explanation of shapes and relationships). (TIF) [file pone.0027286.s002.tif]

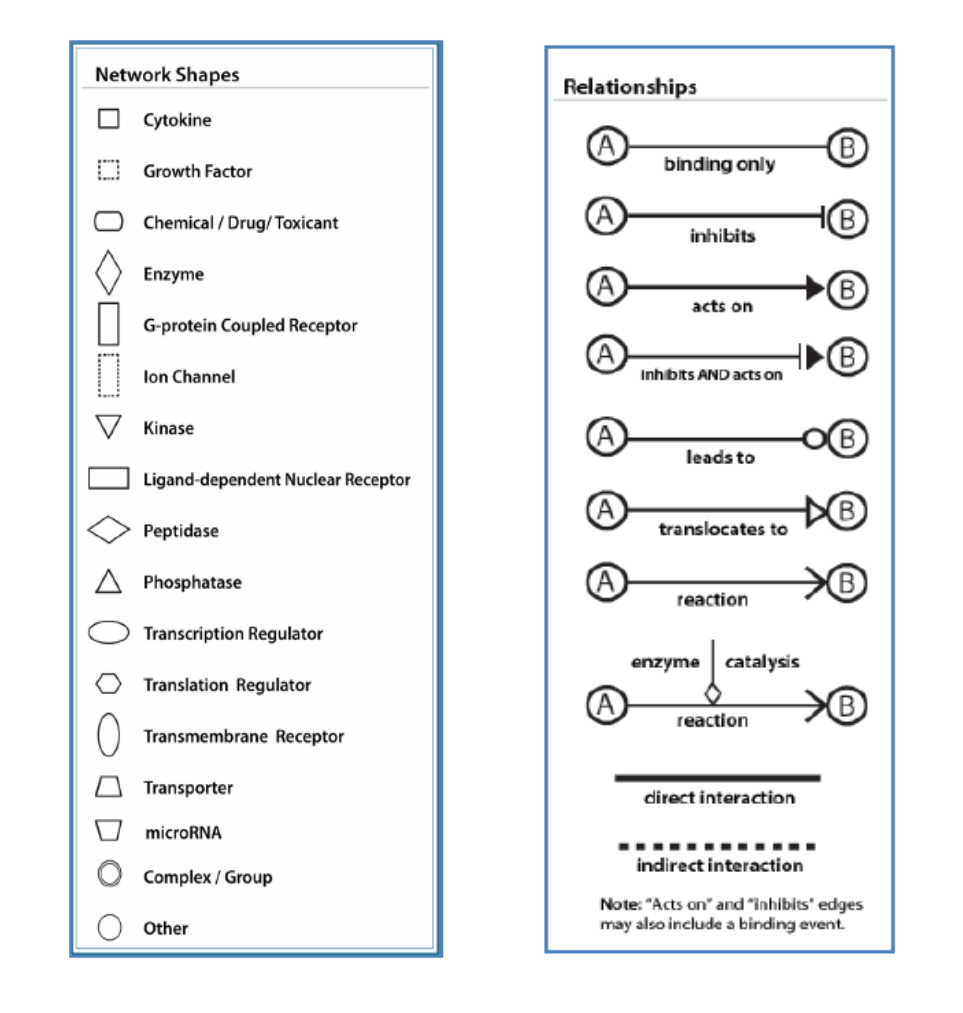

Supplement: Figure S3 — Keys to symbols in networks and pathways. (TIF) [file pone.0027286.s003.tif]
