# Supplementary material for: Epigenetic Mechanism Underlying the Development of Polycystic Ovary Syndrome (PCOS)-Like Phenotypes in Prenatally Androgenized Rhesus Monkeys
Source: PLoS One. 2011 Nov 4;6(11):e27286. doi: 10.1371/journal.pone.0027286 (PMC3208630; doi:10.1371/journal.pone.0027286)
Supplement: Table S1 — Summary of regression analysis to determine whether experimental factors were associated with any principal components in infant data (top table) and adult data (bottom table). P values are displayed in the table. (DOC) [file pone.0027286.s004.doc]

**Table S1**. Summary of regression analysis to determine whether experimental factors were associated with any principal components in infant data (top table) and adult data (bottom table). P values are displayed in the table.

| **Infant EPA (n=7) and control (n=5)** | | | | | |
| --- | --- | --- | --- | --- | --- |
|  | PC1 | PC2 | PC3 | PC4 | PC5 |
| DNA concentration | 0.70 | 0.44 | 0.45 | 0.77 | 0.12 |
| ODs 260/280 | 0.87 | 0.96 | 0.23 | 0.54 | 0.30 |
| **Adult EPA (n=8) and control (n=5)** | | | | | |
|  | PC1 | PC2 | PC3 | PC4 | PC5 |
| DNA concentration | 0.96 | 0.99 | 0.15 | 0.27 | 0.94 |
| ODs 260/280 | 0.076 | 0.19 | 0.39 | 0.82 | 0.12 |
| Plate | 0.12 | 0.13 | 0.57 | 0.73 | 0.38 |
