# Supplementary material for: Epigenetic Mechanism Underlying the Development of Polycystic Ovary Syndrome (PCOS)-Like Phenotypes in Prenatally Androgenized Rhesus Monkeys
Source: PLoS One. 2011 Nov 4;6(11):e27286. doi: 10.1371/journal.pone.0027286 (PMC3208630; doi:10.1371/journal.pone.0027286)
Supplement: Table S2 — 163 significantly differentially methylated genes deemed valid when comparing infant PA and control monkeys. Median and interquartile range (IQR) are presented for control and PA monkeys at each probe. Genes were sorted by BSCVD P values. (DOC) [file pone.0027286.s005.doc]

**Table S2**. 163 significantly differentially methylated genes when comparing infant PA and control monkeys. Median and interquartile range (IQR) are presented for control and PA monkeys at each probe. Genes were sorted by BSCVD P values.

| **Gene Symbol** | **Infant Control** | | **Infant PA** | | **BCSVD P** |
| --- | --- | --- | --- | --- | --- |
|  | **Median-Control** | **IQR-Control** | **Median-PA** | **IQR-PA** |  |
| *MARCH3* | 46.90% | 1.97% | 53.88% | 3.41% | 0.010 |
| *TCEAL6* | 20.70% | 5.20% | 15.00% | 1.49% | 0.010 |
| *KIAA0802* | 85.19% | 1.28% | 84.37% | 1.04% | 0.011 |
| *MYT1* | 37.52% | 5.60% | 44.66% | 2.56% | 0.012 |
| *MDM4* | 4.56% | 1.08% | 3.13% | 0.66% | 0.012 |
| *ELSPBP1* | 52.42% | 5.60% | 59.03% | 3.69% | 0.013 |
| *RHD* | 62.89% | 1.99% | 50.93% | 2.10% | 0.014 |
| *BTK* | 28.72% | 3.16% | 24.39% | 1.87% | 0.014 |
| *CDCA8* | 11.66% | 0.30% | 7.13% | 2.60% | 0.014 |
| *USP34* | 48.03% | 2.49% | 52.85% | 4.10% | 0.015 |
| *RBBP7* | 49.78% | 7.23% | 32.51% | 1.07% | 0.015 |
| *LMBR1L* | 5.73% | 0.21% | 4.82% | 0.65% | 0.016 |
| *C6orf165* | 8.10% | 0.35% | 6.27% | 1.13% | 0.016 |
| *USMG5* | 6.75% | 0.16% | 5.61% | 0.56% | 0.016 |
| *CEP350* | 17.51% | 1.50% | 12.90% | 2.38% | 0.017 |
| *MAPKAPK3* | 5.70% | 1.97% | 3.67% | 0.88% | 0.017 |
| *DAAM2* | 19.45% | 3.33% | 16.30% | 2.68% | 0.017 |
| *SOSTDC1* | 24.59% | 1.73% | 21.28% | 2.46% | 0.018 |
| *C19ORF51* | 8.44% | 0.30% | 7.07% | 0.29% | 0.018 |
| *CSF3R* | 57.69% | 2.53% | 63.42% | 1.87% | 0.018 |
| *GRIA1* | 13.00% | 0.50% | 10.62% | 2.33% | 0.019 |
| *FRZB* | 8.70% | 1.26% | 6.70% | 0.93% | 0.019 |
| *CHMP2B* | 58.01% | 1.87% | 64.10% | 2.08% | 0.019 |
| *CHMP5* | 6.77% | 0.75% | 5.83% | 0.69% | 0.021 |
| *JAKMIP2* | 11.34% | 2.92% | 9.21% | 1.26% | 0.021 |
| *ST13* | 2.80% | 0.32% | 2.42% | 0.05% | 0.021 |
| *RFTN1* | 7.00% | 0.67% | 6.28% | 0.33% | 0.022 |
| *HMGA2* | 5.64% | 0.13% | 4.87% | 0.75% | 0.022 |
| *PLUNC* | 38.71% | 3.12% | 50.06% | 4.53% | 0.022 |
| *ZNF536* | 38.90% | 0.61% | 46.25% | 4.19% | 0.022 |
| *TMEM24* | 5.41% | 0.43% | 6.42% | 0.55% | 0.023 |
| *RYBP* | 8.02% | 1.58% | 10.78% | 1.15% | 0.023 |
| *GLO1* | 5.51% | 0.59% | 4.67% | 0.19% | 0.023 |
| *CTPS* | 3.67% | 0.30% | 3.32% | 0.43% | 0.023 |
| *FAM167B* | 24.46% | 6.34% | 19.63% | 2.50% | 0.024 |
| *PAK3* | 29.77% | 5.60% | 23.53% | 3.19% | 0.024 |
| *LHX6* | 7.50% | 0.98% | 6.92% | 0.49% | 0.025 |
| *BMF* | 4.51% | 0.02% | 4.02% | 0.15% | 0.025 |
| *CILP* | 76.70% | 5.84% | 81.24% | 3.67% | 0.025 |
| *EPHB3* | 4.48% | 0.99% | 3.22% | 0.33% | 0.025 |
| *FBXL19* | 89.71% | 1.52% | 90.30% | 0.81% | 0.026 |
| *MYD88* | 5.52% | 0.80% | 4.44% | 0.79% | 0.026 |
| *MAK3* | 14.01% | 1.89% | 11.41% | 1.55% | 0.026 |
| *RLF* | 6.26% | 1.53% | 3.83% | 0.30% | 0.027 |
| *KIF12* | 41.82% | 6.69% | 36.55% | 1.85% | 0.027 |
| *FHIT* | 6.57% | 1.03% | 5.38% | 0.57% | 0.027 |
| *PDLIM3* | 14.74% | 3.92% | 11.77% | 1.65% | 0.027 |
| *FLRT3* | 18.62% | 2.28% | 16.14% | 2.30% | 0.027 |
| *MFN1* | 9.96% | 0.50% | 8.92% | 0.69% | 0.027 |
| *DZIP1L* | 3.50% | 0.55% | 2.80% | 0.51% | 0.027 |
| *RD3* | 35.17% | 20.55% | 45.41% | 5.77% | 0.028 |
| *CDH6* | 5.85% | 0.23% | 5.25% | 0.37% | 0.028 |
| *TEAD4* | 4.94% | 0.76% | 4.34% | 1.07% | 0.028 |
| *TRIM21* | 6.10% | 0.72% | 4.51% | 1.16% | 0.028 |
| *ITIH2* | 53.40% | 4.37% | 56.67% | 2.50% | 0.028 |
| *PHF13* | 9.66% | 1.43% | 7.67% | 0.45% | 0.029 |
| *FIP1L1* | 45.28% | 15.93% | 49.92% | 1.99% | 0.029 |
| *SESN1* | 8.71% | 0.28% | 6.45% | 1.51% | 0.029 |
| *C21orf33* | 6.77% | 1.42% | 5.19% | 0.84% | 0.029 |
| *DES* | 43.55% | 5.74% | 39.31% | 4.01% | 0.029 |
| *KIAA0174* | 8.52% | 0.85% | 6.72% | 0.69% | 0.029 |
| *GALNT6* | 55.72% | 8.98% | 66.15% | 3.99% | 0.029 |
| *FBXO28* | 4.55% | 0.10% | 5.79% | 1.41% | 0.030 |
| *C10orf6* | 11.51% | 1.07% | 10.03% | 1.13% | 0.030 |
| *C9orf48* | 68.95% | 4.18% | 74.83% | 1.42% | 0.030 |
| *TFF3* | 75.17% | 2.19% | 72.79% | 2.55% | 0.030 |
| *SIX1* | 7.08% | 0.95% | 7.82% | 0.59% | 0.030 |
| *RP2* | 29.06% | 1.90% | 23.51% | 2.80% | 0.031 |
| *C1orf74* | 11.87% | 1.42% | 7.64% | 2.20% | 0.031 |
| *C14orf4* | 4.13% | 0.40% | 3.03% | 0.67% | 0.031 |
| *C6orf105* | 37.71% | 1.11% | 33.75% | 2.58% | 0.031 |
| *DCAF4L2* | 64.73% | 4.72% | 71.22% | 2.33% | 0.031 |
| *PRPF3* | 11.37% | 1.98% | 8.27% | 2.91% | 0.031 |
| *SLC26A10* | 37.65% | 12.18% | 43.00% | 4.53% | 0.032 |
| *IGF2BP2* | 8.65% | 0.59% | 10.28% | 1.12% | 0.032 |
| *RING1* | 11.80% | 0.50% | 10.01% | 1.02% | 0.032 |
| *CNIH4* | 8.06% | 1.69% | 6.32% | 0.73% | 0.032 |
| *ZNF597* | 13.42% | 0.24% | 11.05% | 1.01% | 0.032 |
| *CD55* | 12.01% | 0.61% | 9.51% | 1.37% | 0.032 |
| *ADAMTSL1* | 7.43% | 2.64% | 6.25% | 1.66% | 0.033 |
| *THOC5* | 6.27% | 0.53% | 5.04% | 0.39% | 0.033 |
| *MASP2* | 91.57% | 1.89% | 91.02% | 1.06% | 0.033 |
| *MAT2A* | 3.51% | 0.24% | 3.10% | 0.42% | 0.033 |
| *VPS45A* | 6.17% | 1.82% | 5.26% | 1.38% | 0.034 |
| *RPS10* | 12.16% | 1.13% | 9.79% | 1.28% | 0.034 |
| *CAMP* | 86.02% | 1.45% | 84.79% | 1.23% | 0.034 |
| *OR10H2* | 47.47% | 1.98% | 40.95% | 1.21% | 0.034 |
| *PDCD1LG2* | 21.77% | 6.56% | 16.75% | 2.44% | 0.035 |
| *CDC6* | 6.10% | 0.42% | 5.29% | 0.67% | 0.035 |
| *ZNF192* | 5.69% | 0.70% | 4.87% | 0.28% | 0.035 |
| *TBX19* | 84.41% | 1.95% | 83.53% | 4.01% | 0.035 |
| *HORMAD1* | 71.04% | 3.64% | 75.80% | 1.97% | 0.036 |
| *ZNF512* | 16.21% | 0.96% | 13.37% | 1.37% | 0.037 |
| *MTMR2* | 21.42% | 1.82% | 19.04% | 1.87% | 0.037 |
| *DDB1* | 9.43% | 1.19% | 8.19% | 0.75% | 0.037 |
| *ADAMTS19* | 4.75% | 0.96% | 5.75% | 0.77% | 0.037 |
| *PABPC1* | 13.94% | 0.21% | 12.93% | 1.28% | 0.037 |
| *NRP2* | 8.96% | 0.55% | 7.43% | 0.88% | 0.038 |
| *CAV1* | 5.22% | 0.23% | 5.65% | 0.55% | 0.038 |
| *GDF2* | 80.13% | 11.59% | 70.17% | 3.11% | 0.038 |
| *ELAVL4* | 83.72% | 3.17% | 82.19% | 1.17% | 0.038 |
| *ARPP21* | 69.09% | 4.33% | 75.33% | 2.26% | 0.038 |
| *ETFDH* | 3.86% | 0.26% | 3.04% | 0.88% | 0.039 |
| *NT5C1A* | 14.62% | 0.25% | 13.17% | 0.93% | 0.039 |
| *TREML2* | 52.91% | 4.76% | 59.65% | 2.24% | 0.039 |
| *GPR92* | 60.46% | 2.20% | 55.88% | 4.09% | 0.039 |
| *ANO6* | 9.35% | 1.27% | 6.96% | 0.81% | 0.039 |
| *CYBA* | 4.80% | 0.55% | 4.25% | 0.50% | 0.039 |
| *SMARCAL1* | 73.04% | 1.23% | 74.64% | 2.38% | 0.039 |
| *C7orf23* | 8.12% | 1.35% | 6.33% | 0.91% | 0.039 |
| *IGSF1* | 40.28% | 1.39% | 34.86% | 1.56% | 0.039 |
| *TSPAN31* | 5.01% | 0.71% | 4.34% | 0.57% | 0.039 |
| *POLR2D* | 6.62% | 1.38% | 5.74% | 0.70% | 0.039 |
| *SLC13A2* | 39.16% | 8.41% | 32.69% | 2.48% | 0.039 |
| *ALAS2* | 38.69% | 6.17% | 44.10% | 2.24% | 0.040 |
| *RGR* | 23.54% | 3.27% | 20.99% | 2.86% | 0.040 |
| *MT2A* | 11.31% | 4.78% | 8.46% | 1.91% | 0.040 |
| *SFTPD* | 33.57% | 6.04% | 26.47% | 3.71% | 0.040 |
| *LSR* | 9.21% | 1.10% | 11.12% | 0.86% | 0.041 |
| *FIGNL1* | 4.76% | 1.32% | 3.76% | 0.87% | 0.041 |
| *ZNF235* | 12.20% | 0.49% | 9.35% | 1.59% | 0.041 |
| *CARS* | 9.24% | 1.64% | 11.82% | 1.13% | 0.041 |
| *AMPH* | 4.82% | 1.05% | 5.65% | 0.39% | 0.042 |
| *ATP6V1G1* | 5.42% | 0.22% | 3.92% | 0.51% | 0.042 |
| *SPIN1* | 5.51% | 0.35% | 4.58% | 0.61% | 0.042 |
| *PRKAG2* | 2.25% | 0.60% | 2.80% | 0.32% | 0.042 |
| *GP1BB* | 16.30% | 1.99% | 11.50% | 3.74% | 0.042 |
| *GAS2L1* | 4.25% | 0.22% | 4.90% | 0.83% | 0.043 |
| *SF3B2* | 25.14% | 2.56% | 24.19% | 2.38% | 0.043 |
| *KA35* | 64.90% | 1.17% | 70.13% | 3.52% | 0.043 |
| *CBX6* | 3.70% | 0.47% | 3.34% | 0.23% | 0.044 |
| *DUSP1* | 8.86% | 0.08% | 8.23% | 1.12% | 0.044 |
| *HOXB8* | 9.13% | 0.67% | 7.19% | 0.87% | 0.044 |
| *SMAD4* | 3.57% | 0.72% | 2.94% | 0.42% | 0.044 |
| *TMEM55A* | 4.19% | 0.42% | 3.69% | 0.44% | 0.045 |
| *ARMCX1* | 10.21% | 1.62% | 9.27% | 1.71% | 0.045 |
| *NOP10* | 12.16% | 2.86% | 10.56% | 0.93% | 0.045 |
| *EGLN3* | 4.60% | 0.22% | 4.02% | 0.63% | 0.045 |
| *NUDT22* | 11.88% | 1.46% | 9.90% | 1.17% | 0.045 |
| *GTL3* | 7.77% | 1.15% | 7.12% | 0.87% | 0.045 |
| *LPPR1* | 5.54% | 0.99% | 5.11% | 0.40% | 0.046 |
| *ISYNA1* | 18.24% | 1.11% | 20.14% | 1.46% | 0.046 |
| *IKBIP* | 4.78% | 0.11% | 4.01% | 0.87% | 0.046 |
| *SLAIN1* | 71.54% | 4.55% | 73.93% | 1.42% | 0.046 |
| *NKD2* | 4.27% | 1.02% | 3.49% | 0.47% | 0.046 |
| *FAM83H* | 88.80% | 1.39% | 88.96% | 2.55% | 0.047 |
| *CBS* | 6.62% | 0.74% | 4.90% | 0.45% | 0.047 |
| *SLC20A1* | 2.86% | 0.58% | 2.41% | 0.34% | 0.047 |
| *CDKN1B* | 6.88% | 0.53% | 6.21% | 0.31% | 0.047 |
| *RUSC1* | 6.82% | 0.59% | 6.01% | 0.67% | 0.047 |
| *NTRK3* | 6.86% | 1.13% | 5.47% | 0.63% | 0.048 |
| *RER1* | 4.94% | 1.84% | 2.89% | 0.38% | 0.048 |
| *NPY5R* | 12.13% | 2.06% | 11.40% | 0.75% | 0.048 |
| *PSMD2* | 3.77% | 0.41% | 3.16% | 0.94% | 0.048 |
| *CYP27B1* | 59.33% | 3.26% | 56.25% | 1.78% | 0.048 |
| *SLCO1C1* | 42.06% | 5.40% | 49.16% | 4.48% | 0.048 |
| *CLASP2* | 2.41% | 0.54% | 2.03% | 0.26% | 0.048 |
| *ERCC8* | 4.25% | 0.27% | 2.84% | 0.89% | 0.049 |
| *VPS54* | 12.17% | 0.99% | 10.68% | 0.35% | 0.049 |
| *RAB4A* | 14.49% | 2.83% | 11.72% | 2.52% | 0.049 |
| *MAP3K3* | 3.36% | 0.06% | 3.84% | 0.66% | 0.050 |
| *HSD17B7* | 13.58% | 4.10% | 11.04% | 1.79% | 0.050 |
| *RFX2* | 4.41% | 0.57% | 4.05% | 0.38% | 0.050 |
