# Supplementary material for: Epigenetic Mechanism Underlying the Development of Polycystic Ovary Syndrome (PCOS)-Like Phenotypes in Prenatally Androgenized Rhesus Monkeys
Source: PLoS One. 2011 Nov 4;6(11):e27286. doi: 10.1371/journal.pone.0027286 (PMC3208630; doi:10.1371/journal.pone.0027286)
Supplement: Table S3 — 325 significantly differentially methylated genes deemed valid when comparing adult PA and control monkeys. Median and interquartile range (IQR) are presented for control and PA monkeys at each probe. Genes were sorted by BSCVD P values. (DOC) [file pone.0027286.s006.doc]

**Table S3.** 325 significantly differentially methylated genes deemed valid when comparing adult PA and control monkeys. Median and interquartile range (IQR) are presented for control and PA monkeys at each probe. Genes were sorted by BSCVD P values.

| **Gene Symbol** | **Control Adult** | | **PA Adult** | | **BCSVD P** |
| --- | --- | --- | --- | --- | --- |
|  | **Median-CTL** | **IQR-CTL** | **Median-PA** | **IQR-PA** |  |
| *SCARA5* | 13.18% | 1.27% | 16.21% | 1.05% | 0.003 |
| *ST5* | 76.38% | 2.23% | 70.34% | 6.59% | 0.003 |
| *ZNF541* | 14.92% | 3.11% | 20.03% | 4.23% | 0.005 |
| *IGF2AS* | 15.65% | 2.63% | 25.98% | 6.53% | 0.006 |
| *CRISPLD1* | 7.89% | 0.80% | 10.36% | 2.74% | 0.006 |
| *CSTF3* | 9.59% | 1.65% | 11.91% | 3.24% | 0.006 |
| *B3GNT1* | 3.82% | 0.69% | 4.99% | 1.46% | 0.006 |
| *PPP1R15B* | 3.31% | 0.59% | 2.64% | 1.41% | 0.006 |
| *DNAJC6* | 6.21% | 0.57% | 8.98% | 2.14% | 0.007 |
| *NT5E* | 7.79% | 0.90% | 10.61% | 2.08% | 0.007 |
| *C10orf26* | 7.48% | 0.53% | 10.34% | 2.48% | 0.007 |
| *SIX3* | 7.02% | 0.11% | 7.93% | 0.41% | 0.007 |
| *JPH2* | 9.99% | 2.11% | 14.62% | 4.55% | 0.007 |
| *FANCD2* | 7.85% | 2.24% | 10.47% | 3.90% | 0.007 |
| *CCDC6* | 8.53% | 0.65% | 11.23% | 2.56% | 0.007 |
| *C1orf83* | 4.62% | 1.49% | 10.42% | 4.09% | 0.008 |
| *PFKFB4* | 4.35% | 0.07% | 3.70% | 0.50% | 0.008 |
| *KRTAP15-1* | 77.97% | 4.63% | 73.26% | 2.04% | 0.008 |
| *TFAP2D* | 12.55% | 1.29% | 16.53% | 4.77% | 0.008 |
| *UNC5B* | 4.47% | 0.63% | 6.39% | 1.66% | 0.008 |
| *SNRPB* | 12.64% | 4.71% | 18.32% | 6.70% | 0.009 |
| *IFIH1* | 10.70% | 2.36% | 8.82% | 4.33% | 0.009 |
| *PSME4* | 5.59% | 0.42% | 4.79% | 0.20% | 0.009 |
| *IGF2AS* | 8.47% | 2.73% | 23.26% | 8.93% | 0.010 |
| *GTF3A* | 3.36% | 0.06% | 3.06% | 0.78% | 0.010 |
| *PFDN5* | 4.89% | 1.41% | 7.57% | 2.14% | 0.010 |
| *WNT4* | 24.30% | 0.77% | 27.37% | 3.30% | 0.011 |
| *UCHL1* | 8.54% | 0.58% | 10.29% | 1.40% | 0.011 |
| *KCNH1* | 3.22% | 0.43% | 4.37% | 1.04% | 0.011 |
| *CTSD* | 4.38% | 0.58% | 3.74% | 0.93% | 0.011 |
| *RNF111* | 4.96% | 0.63% | 7.08% | 2.32% | 0.011 |
| *MCM3AP* | 32.83% | 3.04% | 37.56% | 4.97% | 0.011 |
| *DNAJB7* | 83.72% | 3.79% | 79.50% | 8.24% | 0.011 |
| *USF1* | 8.09% | 1.87% | 10.69% | 3.88% | 0.011 |
| *LOC338799* | 7.59% | 1.44% | 10.34% | 2.96% | 0.012 |
| *PCSK2* | 6.39% | 0.25% | 8.49% | 2.81% | 0.013 |
| *DNPEP* | 7.28% | 2.05% | 9.90% | 4.68% | 0.013 |
| *NMUR1* | 42.75% | 1.01% | 39.04% | 2.32% | 0.013 |
| *HBP1* | 2.60% | 0.47% | 2.15% | 0.98% | 0.013 |
| *SKP2* | 7.63% | 0.60% | 11.26% | 4.19% | 0.014 |
| *PLD4* | 31.48% | 4.79% | 28.98% | 8.22% | 0.014 |
| *FTSJD1* | 5.59% | 0.35% | 5.09% | 0.62% | 0.014 |
| *FERD3L* | 7.04% | 1.62% | 9.67% | 2.50% | 0.014 |
| *SCAP1* | 84.24% | 3.03% | 79.17% | 5.02% | 0.014 |
| *CISH* | 2.94% | 0.43% | 2.82% | 0.81% | 0.014 |
| *C9orf139* | 43.89% | 6.27% | 45.38% | 4.88% | 0.014 |
| *ZNF512* | 9.19% | 0.65% | 8.57% | 0.52% | 0.014 |
| *TDRD5* | 7.92% | 0.65% | 12.35% | 4.27% | 0.014 |
| *HKR2* | 2.25% | 0.14% | 3.59% | 1.91% | 0.015 |
| *FLJ21945* | 6.05% | 0.64% | 5.03% | 0.33% | 0.015 |
| *PRKAB2* | 5.66% | 0.88% | 5.09% | 0.37% | 0.015 |
| *LIN10* | 3.77% | 0.48% | 3.26% | 0.26% | 0.015 |
| *TRAF2* | 6.07% | 0.62% | 4.96% | 2.15% | 0.015 |
| *TFAP2E* | 55.25% | 6.87% | 64.88% | 4.69% | 0.015 |
| *ELF4* | 45.24% | 2.21% | 48.24% | 4.57% | 0.015 |
| *SHCBP1* | 7.29% | 3.08% | 10.34% | 3.95% | 0.015 |
| *RUNX3* | 6.54% | 2.54% | 8.56% | 0.90% | 0.015 |
| *PMVK* | 6.87% | 2.33% | 10.20% | 3.63% | 0.015 |
| *TMEM68* | 12.07% | 0.47% | 11.08% | 0.31% | 0.015 |
| *PLEKHA6* | 9.08% | 0.85% | 12.01% | 2.07% | 0.016 |
| *BCL11A* | 13.09% | 2.92% | 16.99% | 3.08% | 0.016 |
| *MYCN* | 5.07% | 0.93% | 6.14% | 0.70% | 0.016 |
| *HOXC8* | 5.58% | 0.68% | 6.83% | 1.64% | 0.016 |
| *TOR3A* | 4.11% | 0.33% | 6.17% | 2.86% | 0.016 |
| *NR4A3* | 3.31% | 0.19% | 4.31% | 0.98% | 0.016 |
| *ITGB3BP* | 6.57% | 0.98% | 5.50% | 2.17% | 0.016 |
| *SLITRK4* | 19.93% | 1.20% | 25.19% | 4.59% | 0.016 |
| *LMX1A* | 4.82% | 0.19% | 6.55% | 2.15% | 0.016 |
| *CHEK2* | 5.53% | 0.91% | 5.14% | 0.48% | 0.016 |
| *PNMA1* | 5.02% | 0.43% | 4.12% | 0.27% | 0.016 |
| *CNTN2* | 23.66% | 4.12% | 25.08% | 2.56% | 0.016 |
| *ZFP42* | 9.65% | 0.66% | 12.87% | 3.38% | 0.016 |
| *SCYL1* | 3.55% | 0.59% | 2.93% | 0.40% | 0.016 |
| *SERBP1* | 7.60% | 0.99% | 6.47% | 0.91% | 0.016 |
| *SLC25A4* | 11.55% | 1.94% | 14.24% | 2.10% | 0.017 |
| *MIOX* | 33.74% | 2.78% | 53.16% | 20.54% | 0.017 |
| *DLX4* | 5.51% | 0.13% | 6.73% | 0.83% | 0.017 |
| *METAP2* | 4.52% | 1.45% | 6.11% | 2.14% | 0.017 |
| *PPHLN1* | 9.99% | 1.58% | 14.30% | 5.14% | 0.017 |
| *AGT* | 30.47% | 2.89% | 33.55% | 3.94% | 0.017 |
| *NUAK2* | 3.73% | 0.51% | 2.91% | 0.92% | 0.017 |
| *ITGA2* | 10.92% | 0.11% | 14.13% | 2.71% | 0.017 |
| *ERH* | 3.92% | 0.26% | 3.60% | 0.41% | 0.017 |
| *CRLF3* | 10.02% | 1.41% | 12.69% | 2.12% | 0.017 |
| *MBNL3* | 74.33% | 2.83% | 69.51% | 6.99% | 0.018 |
| *EIF3D* | 11.69% | 2.06% | 13.42% | 1.28% | 0.018 |
| *CREBL1* | 5.02% | 0.42% | 4.65% | 0.75% | 0.018 |
| *FOXN4* | 4.18% | 0.22% | 5.59% | 1.57% | 0.019 |
| *IGF2* | 3.46% | 0.56% | 5.31% | 1.90% | 0.019 |
| *PID1* | 17.81% | 2.69% | 18.09% | 3.06% | 0.019 |
| *MOSC2* | 22.47% | 10.96% | 7.87% | 3.86% | 0.019 |
| *PEG10* | 55.13% | 1.87% | 52.57% | 2.28% | 0.019 |
| *AP3S1* | 4.58% | 0.64% | 4.52% | 2.40% | 0.019 |
| *ZFP28* | 13.34% | 0.08% | 11.09% | 3.86% | 0.019 |
| *LONP1* | 3.17% | 0.97% | 2.70% | 0.57% | 0.019 |
| *SFRP5* | 8.88% | 1.38% | 7.72% | 2.00% | 0.019 |
| *C20orf117* | 78.55% | 1.37% | 74.44% | 3.94% | 0.019 |
| *PCQAP* | 3.01% | 0.96% | 4.52% | 1.53% | 0.020 |
| *MRO* | 13.55% | 1.36% | 16.56% | 1.83% | 0.020 |
| *FOXD3* | 11.55% | 1.28% | 15.94% | 2.29% | 0.020 |
| *TFCP2* | 3.96% | 0.37% | 3.40% | 0.42% | 0.020 |
| *KRAS* | 7.01% | 1.87% | 6.38% | 0.44% | 0.020 |
| *MYL12B* | 6.25% | 0.38% | 5.20% | 0.91% | 0.020 |
| *SEPT9* | 30.39% | 11.67% | 44.98% | 10.59% | 0.020 |
| *SEL1L3* | 8.73% | 2.87% | 11.22% | 5.21% | 0.020 |
| *KCNB1* | 91.69% | 0.37% | 89.95% | 1.66% | 0.020 |
| *KM-HN-1* | 7.23% | 1.73% | 9.47% | 2.66% | 0.020 |
| *DMXL1* | 4.85% | 0.34% | 3.98% | 0.54% | 0.020 |
| *RAB6A* | 5.23% | 0.53% | 4.54% | 0.61% | 0.020 |
| *C6orf69* | 4.38% | 1.03% | 6.06% | 1.90% | 0.020 |
| *SNTA1* | 8.02% | 1.37% | 10.03% | 1.32% | 0.020 |
| *DEXI* | 4.49% | 0.32% | 4.05% | 0.80% | 0.021 |
| *RIOK1* | 16.12% | 4.34% | 27.73% | 12.93% | 0.021 |
| *PPME1* | 9.78% | 1.19% | 12.94% | 3.49% | 0.021 |
| *NPEPPS* | 5.60% | 1.09% | 8.00% | 3.87% | 0.021 |
| *ATF7* | 4.02% | 0.82% | 5.29% | 1.55% | 0.022 |
| *ARPC3* | 34.56% | 0.26% | 29.93% | 1.25% | 0.022 |
| *ZNF236* | 72.59% | 2.75% | 68.39% | 4.33% | 0.022 |
| *CCND1* | 3.89% | 0.26% | 5.02% | 1.02% | 0.022 |
| *ERBB2* | 6.73% | 1.48% | 8.27% | 0.79% | 0.022 |
| *BTG4* | 13.57% | 1.82% | 17.46% | 3.77% | 0.022 |
| *IREB2* | 1.73% | 0.35% | 1.63% | 0.47% | 0.022 |
| *NOS1* | 6.12% | 1.50% | 4.94% | 0.90% | 0.023 |
| *ZNF791* | 3.88% | 0.88% | 5.33% | 1.72% | 0.023 |
| *TMEM15* | 4.12% | 0.38% | 3.69% | 0.62% | 0.023 |
| *C9orf41* | 5.77% | 0.18% | 4.98% | 0.92% | 0.023 |
| *SYDE1* | 11.38% | 1.79% | 15.08% | 4.27% | 0.023 |
| *ALKBH6* | 6.50% | 0.82% | 8.87% | 3.29% | 0.023 |
| *CAPN9* | 88.82% | 1.08% | 84.28% | 3.06% | 0.024 |
| *MXD1* | 5.18% | 0.39% | 7.42% | 1.68% | 0.024 |
| *SLC3A2* | 5.31% | 1.25% | 8.09% | 3.21% | 0.024 |
| *ASB5* | 73.06% | 3.99% | 71.49% | 8.25% | 0.024 |
| *NCAM1* | 3.96% | 0.37% | 3.52% | 0.31% | 0.024 |
| *ETV6* | 3.20% | 0.36% | 3.07% | 0.54% | 0.024 |
| *ADRB1* | 31.11% | 4.48% | 37.87% | 4.87% | 0.024 |
| *FBXO28* | 3.83% | 0.67% | 4.96% | 1.42% | 0.024 |
| *PLEKHA5* | 5.11% | 0.36% | 6.73% | 1.72% | 0.024 |
| *HOXC9* | 7.72% | 0.72% | 8.61% | 1.07% | 0.025 |
| *ADAM10* | 4.43% | 0.50% | 5.88% | 1.86% | 0.025 |
| *WWP2* | 6.13% | 0.14% | 5.42% | 0.80% | 0.025 |
| *WDR37* | 10.67% | 0.58% | 9.98% | 0.86% | 0.025 |
| *ZMPSTE24* | 6.86% | 0.39% | 6.31% | 0.62% | 0.025 |
| *FARS2* | 3.84% | 0.05% | 5.05% | 1.60% | 0.025 |
| *CYB5A* | 4.03% | 0.39% | 6.07% | 2.45% | 0.025 |
| *TPM3* | 86.10% | 0.86% | 83.92% | 3.16% | 0.026 |
| *TBX5* | 8.39% | 2.27% | 10.00% | 2.65% | 0.027 |
| *STAG2* | 34.86% | 1.44% | 29.75% | 3.86% | 0.027 |
| *PLK1S1* | 4.50% | 0.87% | 4.34% | 0.52% | 0.027 |
| *ZNF524* | 2.42% | 0.05% | 2.25% | 0.23% | 0.028 |
| *COX16* | 3.61% | 0.23% | 3.37% | 0.75% | 0.028 |
| *COL1A1* | 5.21% | 0.51% | 6.26% | 0.96% | 0.028 |
| *CIC* | 16.40% | 0.70% | 18.35% | 1.44% | 0.028 |
| *ZNF512* | 10.49% | 1.77% | 11.10% | 3.92% | 0.028 |
| *ERH* | 5.03% | 1.35% | 6.49% | 3.43% | 0.028 |
| *ZNF365* | 4.32% | 0.55% | 5.66% | 1.60% | 0.028 |
| *NEUROG1* | 5.75% | 0.73% | 7.60% | 1.66% | 0.028 |
| *ADI1* | 2.48% | 0.69% | 2.24% | 1.08% | 0.028 |
| *PRRT1* | 15.79% | 2.31% | 12.63% | 4.16% | 0.029 |
| *MYCBP* | 21.43% | 12.46% | 12.58% | 4.90% | 0.029 |
| *BAMBI* | 11.95% | 0.51% | 10.29% | 1.15% | 0.030 |
| *LEMD3* | 8.24% | 0.06% | 10.42% | 2.82% | 0.030 |
| *MORF4L2* | 26.55% | 1.14% | 22.79% | 2.21% | 0.030 |
| *GRM7* | 14.15% | 2.36% | 18.91% | 3.82% | 0.030 |
| *CROP* | 5.85% | 1.35% | 4.64% | 0.69% | 0.031 |
| *SSPN* | 8.53% | 0.69% | 11.45% | 3.10% | 0.031 |
| *EDEM1* | 4.72% | 0.89% | 4.46% | 1.16% | 0.031 |
| *GPR26* | 16.09% | 0.53% | 23.25% | 2.75% | 0.031 |
| *TFE3* | 42.01% | 4.12% | 46.68% | 5.14% | 0.031 |
| *GLO1* | 4.01% | 0.52% | 5.45% | 2.74% | 0.032 |
| *BET1L* | 2.57% | 0.59% | 2.48% | 0.98% | 0.032 |
| *C20orf133* | 4.91% | 0.68% | 6.41% | 1.61% | 0.032 |
| *TCF4* | 10.69% | 0.49% | 12.21% | 1.03% | 0.032 |
| *HRH1* | 77.37% | 1.55% | 72.98% | 6.19% | 0.032 |
| *NOL4* | 11.59% | 1.18% | 14.18% | 6.41% | 0.032 |
| *HIST1H3I* | 8.64% | 2.91% | 13.02% | 5.71% | 0.032 |
| *SYMPK* | 12.18% | 2.24% | 15.38% | 5.15% | 0.032 |
| *KRT83* | 22.05% | 1.35% | 19.90% | 0.72% | 0.032 |
| *RASL12* | 16.16% | 1.30% | 12.85% | 0.89% | 0.032 |
| *K6IRS4* | 86.82% | 0.83% | 83.90% | 4.14% | 0.033 |
| *OBFC2B* | 13.05% | 1.08% | 14.80% | 2.75% | 0.033 |
| *IPO9* | 8.00% | 0.74% | 7.43% | 0.51% | 0.033 |
| *ENTPD7* | 5.29% | 1.46% | 7.92% | 3.01% | 0.033 |
| *MSX1* | 5.49% | 0.10% | 7.78% | 1.99% | 0.033 |
| *HSP90B1* | 8.25% | 0.66% | 10.28% | 1.51% | 0.033 |
| *BMP2* | 4.40% | 0.64% | 6.02% | 1.42% | 0.033 |
| *PPIH* | 6.07% | 0.41% | 5.75% | 0.80% | 0.034 |
| *TBC1D10C* | 75.92% | 2.93% | 68.25% | 4.52% | 0.034 |
| *ZNF313* | 2.26% | 0.36% | 3.50% | 1.21% | 0.034 |
| *RARB* | 4.32% | 0.63% | 6.48% | 2.18% | 0.034 |
| *OACT2* | 4.83% | 0.59% | 3.86% | 1.10% | 0.034 |
| *PRKAG1* | 3.54% | 0.34% | 3.54% | 0.60% | 0.034 |
| *HCA112* | 5.54% | 1.30% | 7.76% | 0.57% | 0.034 |
| *PPT2* | 11.56% | 3.94% | 6.78% | 2.95% | 0.034 |
| *NEUROD2* | 25.03% | 2.96% | 26.49% | 2.29% | 0.034 |
| *TINAG* | 50.87% | 5.36% | 44.36% | 12.61% | 0.035 |
| *MLF1* | 2.15% | 0.59% | 3.23% | 1.35% | 0.035 |
| *RBM12* | 4.21% | 1.11% | 3.75% | 0.51% | 0.035 |
| *TGFBR1* | 5.81% | 0.97% | 8.11% | 2.22% | 0.035 |
| *DAXX* | 4.17% | 1.33% | 5.99% | 2.69% | 0.035 |
| *STIM1* | 4.51% | 0.57% | 5.66% | 1.93% | 0.035 |
| *KCNH4* | 7.35% | 0.89% | 10.02% | 1.43% | 0.035 |
| *NARG1* | 2.53% | 0.57% | 2.19% | 0.63% | 0.035 |
| *C8orf37* | 3.99% | 0.66% | 5.02% | 1.91% | 0.035 |
| *MRRF* | 4.09% | 0.45% | 3.72% | 0.76% | 0.036 |
| *PCDHA9* | 46.91% | 0.80% | 50.65% | 3.71% | 0.036 |
| *KLF12* | 4.85% | 1.76% | 8.34% | 5.24% | 0.036 |
| *DEF6* | 15.04% | 1.06% | 11.44% | 1.43% | 0.036 |
| *FKSG44* | 4.35% | 0.79% | 5.33% | 1.53% | 0.037 |
| *TFAP2B* | 15.66% | 2.89% | 18.86% | 8.79% | 0.037 |
| *TBX2* | 26.73% | 3.40% | 30.83% | 3.34% | 0.037 |
| *LYL1* | 9.92% | 1.63% | 15.15% | 3.97% | 0.037 |
| *PFDN5* | 22.44% | 0.55% | 24.35% | 2.02% | 0.037 |
| *APBB1* | 3.51% | 0.68% | 5.26% | 2.62% | 0.038 |
| *SLC24A3* | 3.97% | 0.12% | 5.64% | 1.69% | 0.038 |
| *PAK3* | 8.02% | 1.01% | 9.71% | 1.58% | 0.038 |
| *ZNF710* | 2.50% | 0.60% | 3.15% | 1.11% | 0.038 |
| *TOPORS* | 6.76% | 2.32% | 10.17% | 5.60% | 0.038 |
| *SLITRK1* | 6.96% | 1.56% | 9.98% | 3.78% | 0.038 |
| *DNMT3A* | 5.77% | 0.59% | 5.23% | 1.07% | 0.038 |
| *MYL3* | 86.54% | 1.67% | 83.60% | 2.66% | 0.038 |
| *MYO18A* | 6.31% | 0.75% | 8.22% | 2.73% | 0.039 |
| *PRUNE* | 4.11% | 0.29% | 4.01% | 0.53% | 0.039 |
| *SERPING1* | 9.24% | 2.44% | 11.83% | 3.95% | 0.039 |
| *CPSF3* | 4.02% | 1.23% | 3.50% | 0.44% | 0.039 |
| *MYCL1* | 3.97% | 0.14% | 5.39% | 1.99% | 0.039 |
| *SOCS3* | 4.29% | 0.31% | 3.80% | 0.74% | 0.039 |
| *SSPN* | 10.12% | 0.12% | 12.09% | 2.56% | 0.039 |
| *ESPN* | 13.11% | 15.29% | 25.82% | 2.08% | 0.039 |
| *SOX2* | 7.47% | 1.85% | 9.38% | 1.69% | 0.040 |
| *PRKD1* | 41.11% | 15.46% | 53.21% | 5.12% | 0.040 |
| *ICMT* | 3.73% | 0.52% | 3.19% | 0.37% | 0.040 |
| *AMID* | 3.07% | 0.62% | 4.15% | 1.03% | 0.040 |
| *DNAJC7* | 10.99% | 0.77% | 14.63% | 1.77% | 0.040 |
| *AIM1* | 4.13% | 0.43% | 3.41% | 0.19% | 0.040 |
| *SH3GL3* | 3.57% | 0.33% | 5.86% | 1.49% | 0.040 |
| *TCL1A* | 77.50% | 0.38% | 72.22% | 4.09% | 0.040 |
| *C1D* | 21.68% | 10.30% | 25.17% | 11.88% | 0.040 |
| *LOC89944* | 6.05% | 1.48% | 9.25% | 3.65% | 0.040 |
| *FLJ10786* | 9.31% | 2.09% | 7.22% | 2.30% | 0.041 |
| *KCNA4* | 6.71% | 1.42% | 8.57% | 1.15% | 0.041 |
| *CYC1* | 3.73% | 0.52% | 3.55% | 0.81% | 0.041 |
| *HMBS* | 3.80% | 0.53% | 3.37% | 0.40% | 0.041 |
| *SLC35F2* | 4.62% | 0.24% | 6.83% | 2.71% | 0.041 |
| *EGR2* | 3.15% | 0.12% | 4.23% | 0.89% | 0.041 |
| *CYB5-M* | 5.56% | 1.13% | 7.06% | 1.88% | 0.041 |
| *ZDHHC19* | 82.72% | 4.13% | 85.39% | 2.79% | 0.041 |
| *RHPN2* | 5.04% | 0.44% | 7.84% | 2.72% | 0.041 |
| *TIMM9* | 3.37% | 0.78% | 4.68% | 2.10% | 0.041 |
| *SFRP1* | 4.88% | 0.48% | 6.56% | 1.06% | 0.041 |
| *ZNF524* | 3.54% | 0.63% | 5.36% | 1.87% | 0.041 |
| *EIF5A* | 6.48% | 1.34% | 8.67% | 2.84% | 0.042 |
| *ALKBH4* | 7.03% | 0.23% | 9.07% | 2.46% | 0.042 |
| *MAGI2* | 4.38% | 0.25% | 5.92% | 1.65% | 0.042 |
| *TDRD7* | 11.54% | 0.30% | 10.92% | 1.48% | 0.042 |
| *SP140* | 90.71% | 1.18% | 89.04% | 2.16% | 0.042 |
| *SUV39H2* | 3.29% | 1.02% | 2.99% | 0.67% | 0.042 |
| *F2R* | 8.06% | 4.05% | 7.17% | 1.75% | 0.042 |
| *MTSS1* | 72.60% | 17.33% | 44.76% | 12.10% | 0.042 |
| *KIT* | 14.95% | 1.62% | 22.51% | 9.27% | 0.042 |
| *EMILIN1* | 41.33% | 5.71% | 46.66% | 3.05% | 0.042 |
| *SFRS2* | 17.88% | 2.73% | 15.52% | 1.49% | 0.042 |
| *MYOD1* | 7.40% | 0.71% | 9.07% | 0.84% | 0.042 |
| *NUMBL* | 3.60% | 0.59% | 5.01% | 1.60% | 0.042 |
| *RRAD* | 10.82% | 0.65% | 9.75% | 2.00% | 0.042 |
| *ADAR* | 11.04% | 3.25% | 9.97% | 1.33% | 0.043 |
| *KCNF1* | 7.53% | 0.79% | 8.74% | 1.96% | 0.043 |
| *ZNF286* | 13.37% | 1.66% | 16.22% | 4.97% | 0.043 |
| *ANTXR1* | 3.81% | 0.40% | 4.92% | 0.61% | 0.043 |
| *FAM96A* | 5.32% | 0.87% | 4.95% | 0.88% | 0.043 |
| *KLK10* | 6.70% | 0.38% | 6.24% | 0.57% | 0.043 |
| *CCDC3* | 10.81% | 1.79% | 13.58% | 2.56% | 0.043 |
| *MITF* | 4.62% | 0.22% | 6.34% | 2.50% | 0.043 |
| *DLL1* | 6.94% | 0.41% | 8.67% | 2.75% | 0.043 |
| *BRD8* | 3.82% | 0.47% | 3.42% | 0.35% | 0.043 |
| *ALKBH3* | 4.88% | 1.79% | 4.47% | 1.78% | 0.043 |
| *NEK6* | 14.66% | 1.31% | 14.02% | 0.96% | 0.043 |
| *ZFYVE19* | 84.45% | 3.96% | 83.65% | 7.36% | 0.043 |
| *FOXC2* | 6.43% | 1.07% | 5.79% | 1.93% | 0.044 |
| *NID2* | 5.04% | 0.84% | 7.16% | 2.17% | 0.044 |
| *TMEM179* | 13.42% | 0.75% | 15.84% | 1.63% | 0.044 |
| *EFNB1* | 28.58% | 0.87% | 32.91% | 2.62% | 0.044 |
| *NEUROD1* | 7.23% | 1.55% | 8.79% | 0.74% | 0.044 |
| *BNIP2* | 5.12% | 1.70% | 3.63% | 0.54% | 0.044 |
| *CCDC37* | 6.70% | 1.18% | 8.53% | 1.43% | 0.044 |
| *PRKRIR* | 26.65% | 5.27% | 13.85% | 3.48% | 0.045 |
| *RLF* | 3.95% | 0.89% | 5.82% | 2.31% | 0.045 |
| *KIF1C* | 2.80% | 0.39% | 2.19% | 0.93% | 0.045 |
| *MRPL9* | 4.45% | 0.81% | 7.06% | 1.51% | 0.045 |
| *SLC5A2* | 80.93% | 2.07% | 78.21% | 4.72% | 0.045 |
| *GPR133* | 15.23% | 1.71% | 21.03% | 3.35% | 0.045 |
| *SOCS2* | 4.11% | 0.19% | 3.63% | 1.01% | 0.046 |
| *COX6B1* | 7.25% | 0.47% | 10.86% | 5.91% | 0.046 |
| *ZNF668* | 7.07% | 1.75% | 8.34% | 2.82% | 0.046 |
| *MAML2* | 2.61% | 0.57% | 2.44% | 0.51% | 0.046 |
| *KCNK17* | 18.15% | 0.67% | 16.58% | 0.95% | 0.046 |
| *ARMCX1* | 14.44% | 1.10% | 17.90% | 4.59% | 0.046 |
| *TGFB3* | 22.73% | 0.18% | 20.17% | 3.55% | 0.046 |
| *PITX3* | 5.30% | 0.55% | 6.95% | 1.75% | 0.046 |
| *GUCY2C* | 77.23% | 2.58% | 72.23% | 3.42% | 0.046 |
| *GLB1L* | 2.75% | 0.44% | 2.92% | 1.08% | 0.047 |
| *BTBD6* | 34.41% | 1.69% | 40.32% | 4.68% | 0.047 |
| *KCNQ5* | 2.77% | 0.74% | 3.32% | 0.86% | 0.047 |
| *GPS2* | 6.36% | 0.30% | 7.90% | 1.20% | 0.047 |
| *ZNF436* | 3.75% | 0.61% | 3.57% | 0.57% | 0.047 |
| *AR* | 32.12% | 4.99% | 29.88% | 1.77% | 0.047 |
| *RAB25* | 63.17% | 3.83% | 68.04% | 3.77% | 0.047 |
| *SFRS8* | 6.92% | 0.58% | 8.54% | 1.67% | 0.047 |
| *TMEM39A* | 5.12% | 1.14% | 7.49% | 3.69% | 0.048 |
| *PRKAG2* | 7.86% | 0.14% | 6.59% | 1.98% | 0.048 |
| *ATP10A* | 5.22% | 0.88% | 4.43% | 1.55% | 0.048 |
| *OTP* | 6.25% | 0.78% | 7.81% | 1.60% | 0.048 |
| *EPHA5* | 7.04% | 0.58% | 9.45% | 2.56% | 0.048 |
| *NCAM1* | 6.72% | 0.33% | 8.57% | 2.08% | 0.048 |
| *ZNF365* | 4.52% | 0.70% | 3.79% | 0.29% | 0.048 |
| *ARL6IP6* | 2.39% | 0.18% | 2.02% | 0.79% | 0.048 |
| *LOC283537* | 2.99% | 0.88% | 2.71% | 0.59% | 0.048 |
| *PACS1* | 8.47% | 1.09% | 10.98% | 2.30% | 0.048 |
| *TIPARP* | 1.76% | 0.27% | 1.48% | 0.41% | 0.048 |
| *LRRC39* | 87.00% | 1.24% | 83.61% | 4.97% | 0.048 |
| *FOXB1* | 9.02% | 3.38% | 9.96% | 2.30% | 0.048 |
| *NR1H2* | 3.83% | 0.42% | 3.47% | 0.40% | 0.048 |
| *CCPG1* | 7.97% | 1.67% | 5.99% | 0.82% | 0.049 |
| *HERC1* | 10.56% | 0.97% | 8.95% | 1.19% | 0.049 |
| *CYB561D1* | 3.31% | 0.30% | 3.01% | 0.59% | 0.049 |
| *ZNF672* | 2.44% | 0.48% | 2.15% | 0.93% | 0.050 |
