# Supplementary material for: Epigenetic Mechanism Underlying the Development of Polycystic Ovary Syndrome (PCOS)-Like Phenotypes in Prenatally Androgenized Rhesus Monkeys
Source: PLoS One. 2011 Nov 4;6(11):e27286. doi: 10.1371/journal.pone.0027286 (PMC3208630; doi:10.1371/journal.pone.0027286)
Supplement: Table S4 — Full names of genes in text. (DOC) [file pone.0027286.s007.doc]

**Table S4**: Full names of genes in text.

| Gene Symbol | Full Name |
| --- | --- |
| *AKT* | v-akt murine thymoma viral oncogene homolog 1 |
| *AKT2* | v-akt murine thymoma viral oncogene homolog 2 |
| *AMID* | apoptosis-inducing factor, mitochondrion-associated, 2 |
| *AR* | androgen receptor |
| *BMP2* | bone morphogenetic protein 2 |
| *BRD8* | bromodomain containing 8 |
| *CCND1* | cyclin D1 |
| *Creb* | cyclic-AMP response element binding protein |
| *ERBB2* | v-erb-b2 erythroblastic leukemia viral oncogene homolog 2 |
| *ERK* | mitogen-activated protein kinase 1 |
| *FBXO28* | F-box protein 28 |
| *FLJ10786* | coiled-coil domain containing 87 |
| *FSH* | follicle stimulating hormone |
| *hCG* | chorionic gonadotropin, beta polypeptide 5 |
| *HOXC8* | homeobox C8 |
| *JNK* | mitogen-activated protein kinase 8 |
| *KCNQ5* | potassium voltage-gated channel, KQT-like subfamily, member 5 |
| *KRAS* | v-Ki-ras2 Kirsten rat sarcoma viral oncogene homolog |
| *LH* | luteinizing hormone |
| *LRRC39* | leucine rich repeat containing 39 |
| *MORF4L2* | mortality factor 4 like 2 |
| *MYCBP* | c-myc binding protein |
| *NEUROG1* | neurogenin 1 |
| *NFκB* | nuclear factor kappa-B |
| *OACT2* | membrane bound O-acyltransferase domain containing 2 |
| *OBFC2B* | oligonucleotide/oligosaccharide-binding fold containing 2B |
| *P38MAPK* | mitogen-activated protein kinase 14 |
| *P27K1P1* | cyclin-dependent kinase inhibitor 1B (p27, Kip1) |
| *PABP1* | poly(A) binding protein, cytoplasmic 1 |
| *PFKFB4* | 6-phosphofructo-2-kinase/fructose-2,6-biphosphatase 4 |
| *PRKD1* | protein kinase D1 |
| *PSME4* | proteasome (prosome, macropain) activator subunit 4 |
| *RAB6A* | member RAS oncogene family |
| *RRAD* | Ras-related associated with diabetes |
| *RUNX3* | runt-related transcription factor 3 |
| *SEPT9* | septin 9 |
| *SMAD2/3* | SMAD family member 2 and 3 |
| *SMAD4* | SMAD family member 4 |
| *SMAD5* | SMAD family member 5 |
| *SUV39H2* | suppressor of variegation 3-9 homolog 2 (Drosophila) |
| *TBX2* | T-box 2 |
| *TFE3* | transcription factor binding to IGHM enhancer 3 |
| *TGFBR1* | transforming growth factor, beta receptor 1 |
| *ZNF236* | zinc finger protein 236 |
| *ZNF512* | zinc finger protein 512 |
