# Supplementary material for: Epigenetic Mechanism Underlying the Development of Polycystic Ovary Syndrome (PCOS)-Like Phenotypes in Prenatally Androgenized Rhesus Monkeys
Source: PLoS One. 2011 Nov 4;6(11):e27286. doi: 10.1371/journal.pone.0027286 (PMC3208630; doi:10.1371/journal.pone.0027286)
Supplement: Table S5 — PANTHER pathway analysis generated with the Human Methylation27 genes as the reference list. (DOC) [file pone.0027286.s008.doc]

**Table S5**. PANTHER pathway analysis generated with the Human Methylation27 genes as the reference list.

| **Pathway** | **Reference list from array** | **Numbers of differentially methylated genes** | **Expected gene numbers** | **Over(+)/ under(-) representation** | **P-value** |
| --- | --- | --- | --- | --- | --- |
| **Infant** |  |  |  |  |  |
| Salvage pyrimidine ribonucleotides | 15 | 2 | 0.18 | + | 0.014 |
| Cysteine biosynthesis | 2 | 1 | 0.02 | + | 0.024 |
| Oxidative stress response | 53 | 3 | 0.63 | + | 0.026 |
| S adenosyl methionine biosynthesis | 3 | 1 | 0.04 | + | 0.035 |
| **Adult** |  |  |  |  |  |
| TGF-beta signaling pathway | 145 | 9 | 3.26 | + | 0.0061 |
| PI3 kinase pathway | 111 | 7 | 2.5 | + | 0.014 |
| Angiogenesis | 207 | 10 | 4.66 | + | 0.020 |
| p53 pathway by glucose deprivation | 28 | 3 | 0.63 | + | 0.026 |
| Interferon-gamma signaling pathway | 30 | 3 | 0.68 | + | 0.031 |
| Insulin/IGF pathway-protein kinase B signaling cascade | 82 | 5 | 1.85 | + | 0.039 |
| Methylcitrate cycle | 2 | 1 | 0.05 | + | 0.044 |
