# Supplementary material for: Epigenetic Mechanism Underlying the Development of Polycystic Ovary Syndrome (PCOS)-Like Phenotypes in Prenatally Androgenized Rhesus Monkeys
Source: PLoS One. 2011 Nov 4;6(11):e27286. doi: 10.1371/journal.pone.0027286 (PMC3208630; doi:10.1371/journal.pone.0027286)
Supplement: Table S6 — The highest scoring molecule networks in PA infant and adult female monkeys. * = involved in TGF-β signaling; ** = involved in reproduction; *** = involved in adipogenesis. (DOC) [file pone.0027286.s009.doc]

**Table S6.** The highest scoring molecule networks in PA infant and adult female monkeys.

| **Network** | **Score** | **Focus Molecules** | **Top Functions** | **Molecules in Network** |
| --- | --- | --- | --- | --- |
| Infant | 38 | 23 | Nutritional Disease, DNA Replication, Recombination and Repair, Inflammatory Response | Akt***, BTK, cAMP***, CAV1, CD55, CDC6***, CDKN1B***, CREB***, CYP27B1***, DUSP1***, E2f***, ERCC8, ERK***, ERK1/2*/***, FSH**, GAS2L1, hCG**/***, Histone h3, HMGA2***, HOXB8, Jnk***, LH**, MAP3K3, MARCH3, MT2A***, MYD88***, P38 MAPK***, PDLIM3, POLR2D, RAB4A, RNA polymerase II***, SF3B2, SLC20A1, SMAD4*/***, TFF3 |
| Adult | 31 | 23 | Cell Cycle, Cellular Growth and Proliferation, Gene Expression | AGT, Akt***, Ap1***, AR**/***, BMP2*/***, CCND1***, COL1A1***, Collagen type I, Creb***, ERBB2***, ERK*/***, ERK1/2*/***, F2R, hCG**/***, HSP90B1, IGF2***, ITGA2, Laminin***, MSX1***, MXD1***, MYOD1***, NFkB (complex)***, NR1H2***, NR4A3***, PI3K***, Pkc(s)***, PRKD1, RARB***, SIX3, SKP2***, Smad2/3*/***, TGFBR1*/***, TRAF2***, USF1***, WNT4*** |

* involved in TGF-β signaling

** involved in reproduction

*** involved in adipogenesis
